# Relationship of arterial tonometry and exercise in patients with chronic heart failure: a systematic review with meta-analysis and trial sequential analysis

**DOI:** 10.1186/s12872-022-02792-6

**Published:** 2022-08-01

**Authors:** Xiaodan Gong, Mengwen Hu, Mei Li

**Affiliations:** 1 Department of Cardiology, Corporate Member of Freie Universität Berlin and Humboldt-Universität zu Berlin, Charité – Universitätsmedizin Berlin, Augustenburger Platz 1, 13353 Berlin, Germany; 2 Department of Experimental Surgery, Corporate Member of Freie Universität Berlin and Humboldt-Universität zu Berlin, Charité – Universitätsmedizin Berlin, Augustenburger Platz 1, 13353 Berlin, Germany; 3 Institute of Physiology, Corporate Member of Freie Universität Berlin and Humboldt-Universität zu Berlin, Campus Charité Mitte, Charité – Universitätsmedizin Berlin, Charitéplatz 1, 10117 Berlin, Germany

**Keywords:** Arterial stiffness, Arterial tonometry, Exercise, Chronic heart failure, Meta-analysis

## Abstract

**Background:**

Arterial stiffness is a common characteristic in patients with chronic heart failure (CHF), and arterial tonometric technologies related to arterial stiffness are novel and effective methods and have an important value in the diagnosis and prognosis of CHF. In terms of ameliorating arterial stiffness in patients with CHF, exercise training is considered an adjuvant treatment and also an effective means in the diagnosis and judgment of prognosis. However, there are huge controversies and inconsistencies in these aspects. The objective of this meta-analysis was to systematically test the connection of arterial tonometry and exercise in patients with CHF.

**Methods:**

Databases, including MEDLINE, EMBASE, and Cochrane Central Register of Controlled Trials (CENTRAL) in The Cochrane Library, were accessed from inception to 7 March 2022. The meta-analysis was then conducted, and trial sequential analysis (TSA) was performed jointly to further verify our tests and reach more convincing conclusions by using RevMan version 5.4 software, STATA version 16.0 software, and TSA version 0.9.5.10 Beta software.

**Results:**

Eighteen articles were included, with a total of 876 participants satisfying the inclusion criteria. The pooling revealed that flow-mediated dilation (FMD) was lower in basal condition [standardized mean difference (SMD): − 2.28%, 95% confidence interval (CI) − 3.47 to − 1.08, *P* < 0.001] and improved significantly after exercise (SMD: 5.96%, 95% CI 2.81 to 9.05, *P* < 0.001) in patients with heart failure with reduced ejection fraction (HFrEF) compared with healthy participants. The high-intensity training exercise was more beneficial (SMD: 2.88%, 95% CI 1.78 to 3.97, *P* < 0.001) than the moderate-intensity training exercise to improve FMD in patients with CHF. For augmentation index (AIx), our study indicated no significant differences (SMD: 0.50%, 95% CI − 0.05 to 1.05, *P* = 0.074) in patients with heart failure with preserved ejection fraction (HFpEF) compared with healthy participants. However, other outcomes of our study were not identified after further verification using TSA, and more high-quality studies are needed to reach definitive conclusions in the future.

**Conclusions:**

This review shows that FMD is lower in basal condition and improves significantly after exercise in patients with HFrEF compared with healthy population; high-intensity training exercise is more beneficial than moderate-intensity training exercise to improve FMD in patients with CHF; besides, there are no significant differences in AIx in patients with HFpEF compared with the healthy population. More high-quality studies on this topic are warranted.

**Supplementary Information:**

The online version contains supplementary material available at 10.1186/s12872-022-02792-6.

## Introduction

Human senescence has ever-increasing characteristics of arterial stiffness [1–5], and arterial stiffness is a common characteristic in patients with chronic heart failure (CHF) [6–8]. Nowadays, arterial stiffness is commonly considered a predictor of CHF [9–11]. Increased arterial stiffness increases pulsatile load and amplifies pulse pressure which result in the increase of myocardial oxygen consumption in patients with CHF [12, 13]. The identification of arterial tonometric technologies related to arterial stiffness and therapies aiming at decreasing arterial stiffness are novel and effective methods to treat and prevent CHF [13, 14]. So far, there have been a lot of studies about the indexes related to arterial stiffness, however, considerable controversies and large inconsistencies exist among these studies [11, 15–20]. This highlights the significance of integrating and analyzing the related data to reach compelling conclusions of correlation on these tonometric technologies between patients with CHF and healthy population.

CHF is a complicated pathophysiological syndrome, and the most noticeable symptom of patients with CHF is reduced exercise performance and intolerance, especially in patients with severe CHF [21–26]. Exercise training is also regarded as a diagnostic and prognostic means, meanwhile, it can improve exercise performance and intolerance and is considered adjuvant therapy in patients with CHF [27–30]. During exercise, there is relative arterial dilation as the normal reaction for meeting increased metabolic needs [29–35]. Consequently, regular exercise training can increase the capacity of arterial dilation and compliance, and ameliorate arterial stiffness in patients with CHF [36–38]. Despite these advantages, the recommendation of systematical exercise training among patients with CHF has been poorly fulfilled, and there are also inconsistencies regarding the exercise intensity that can produce the optimal effects in available studies. Moderate-intensity training (MIT) exercise is recommended to be applied in patients with CHF in existing guidelines [39–41]. However, some data showed high-intensity training (HIT) exercise may have an advantage over MIT to improve cardiovascular outcomes in patients with CHF [42–44]. There are limited studies assessing the role of exercise in those patients, and there is also a lack of studies to systematically combine arterial tonometric technologies with exercise training in patients with CHF.

For the above reasons, the aims of this study were to systematically test the connection of arterial tonometry between patients with CHF and healthy population and detect the roles that exercise plays in the relationship between arterial tonometry and patients with CHF. In most studies, however, the qualitative findings were limited by the relatively small sample sizes. Meta-analysis has the merit of integrating the studies to draw more persuasive conclusions, therefore, we performed our study using meta-analysis and combined trial sequential analysis (TSA) to further verify our tests and reach more convincing conclusions.

## Methods

### Protocol and guidance

This study was performed following the Preferred Reporting Items for Systematic Reviews and Meta-Analyses (PRISMA) and the Cochrane Handbook for Interventional Reviews [45–47].

### Eligibility criteria

First, only randomized controlled trials (RCTs) were covered in this meta-analysis to ensure consistency and compelling pooled estimates. Second, studies had enrolled patients (aged ≥ 18 years) with CHF were considered, including heart failure with reduced ejection fraction (HFrEF) and heart failure with preserved ejection fraction (HFpEF). Third, studies had used a tonometric assessment of arterial stiffness. Fourth, patients with CHF undergoing HIT or MIT were considered.

### Search strategy

The following electronic databases were searched from inception to 7 March 2022: MEDLINE, EMBASE, and Cochrane Central Register of Controlled Trials (CENTRAL) in The Cochrane Library. To maximize the search of related articles, we reviewed the reference lists of eligible studies and systematic reviews. Our study was limited to RCT, and no language or other limitations were applied. The whole search strategies of all the electronic databases are presented in Additional file 1.

### Study selection

Two authors independently checked all the titles and abstracts and determined possibly relevant studies to be retrieved. When there were uncertainties or disagreements, the full text of the studies was further reviewed. Any disagreements were addressed by consensus, and decisions were checked by a third author.

### Data extraction and management

Two authors used a predefined Excel form to extract data from the included articles. When an article referred to an outcome of interest but without publishing, we contacted the authors for the data. If the authors did not reply, we excluded the articles. All discrepancies were addressed by discussion, and the decisions were checked by a third author.

### Quality assessment

Study risk of bias was assessed by two authors using the Cochrane Collaboration risk of bias tool in RevMan version 5.4 software [47]. The included studies were ranked high risk, unclear, or low risk of bias. If any disagreements occurred, a third author was consulted.

### Statistical analysis

Statistical analyses were performed using STATA version 16.0 software. This meta-analysis has been conducted based on the Preferred Reporting Items for Systematic reviews and Meta-Analyses (PRISMA) guidelines [46]. We used the chi-square test and the I^2^ statistic to evaluate heterogeneity among the studies. If heterogeneity was significant (I^2^ ≥ 50%), we obtained the pooled estimate using the random-effects model, while if heterogeneity was not significant (I^2^ < 50%), we used a fixed-effects model. Given variables in our study were all continuous data, we used the standardized mean difference (SMD) and 95% confidence interval (CI) to assess outcomes. *P*-value less than 0.05 was considered to be statistically significant. Two subgroup analyses were adopted to test interactions based on types of CHF (HFpEF and HFrEF) and exercise intensity (MIT and HIT). Random effects meta-regression was performed to test the correlation between the basic factors and study outcomes. Covariates included overall risk of bias (wherein low bias meant “low risk” occurred ≥ 5 of 8 items, and high bias signified “low risk” < 5 of 8 items), demographic factors (gender and mean age), and basic information (sample size and publication date).

If the continuous data were published as median and interquartile range (IQR) in the included articles, the median was considered to be equal to the mean, and the standard deviation (SD) was calculated roughly based on the formula SD = IQR/1.35 [47]. While if the data were provided as mean and standard error (SE), SD could be calculated by multiplying SE by the square root of the sample size: SD = SE $\sqrt{\mathrm{N}}$.[47]

### Trial sequential analysis

TSA version 0.9.5.10 Beta software was used to analyze whether cumulative data were compelling enough to assess outcomes and was also applied to obtain the required information size (RIS). TSA was performed by an overall type I error of 5% and a power of 80%.

## Results

### Search results

**Fig. 1:**
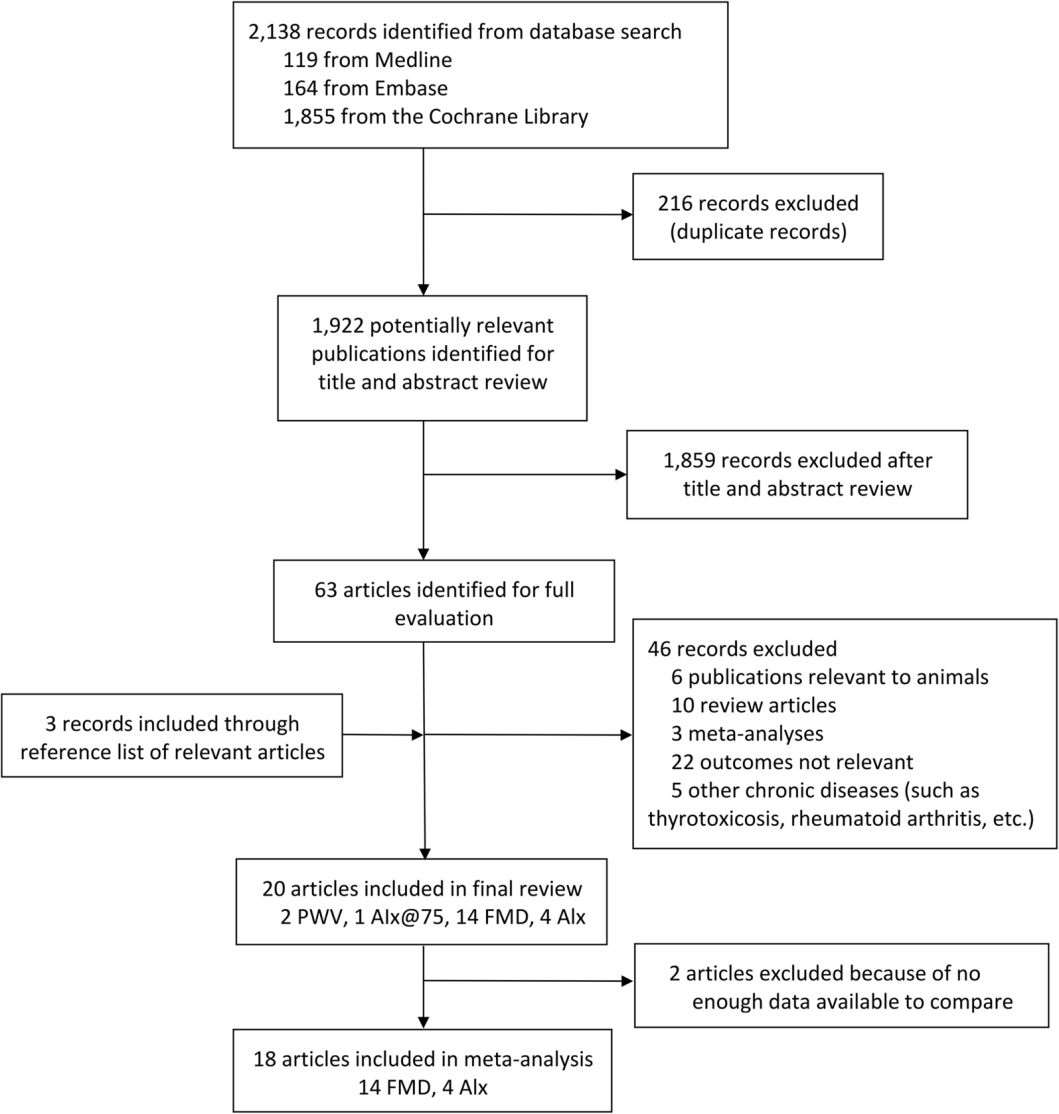
Flow diagram for inclusion of studies in meta-analysis

We identified 63 possibly eligible records from the 2138 articles retrieved from Medline, Embase, and The Cochrane Library. After reviewing the whole contents of the records, 18 articles [20, 21, 25, 38, 42, 44, 48–59] were determined in the final meta-analysis. The process of searching the eligible articles is summarized in Fig. 1.

Four studies involved augmentation index (AIx), and 14 studies involved FMD, wherein four studies compared AIx between patients with CHF and healthy participants, seven studies compared FMD between patients with CHF and healthy participants, two studies compared FMD in patients with CHF on different exercise intensities, and six studies compared FMD in patients with CHF in the cases of exercise and non-exercise.

**Fig. 2:**
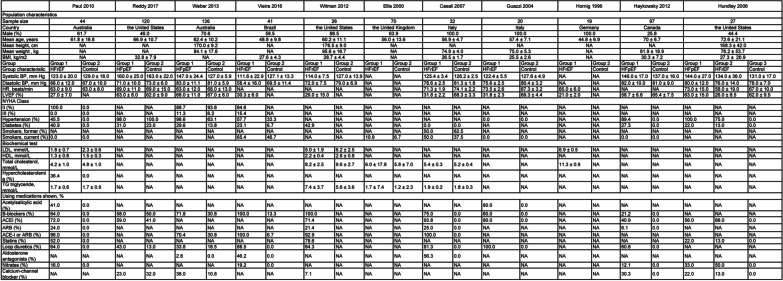
Population characteristics of included studies comparing flow-mediated dilation and augmentation index in patients with chronic heart failure and healthy participants

**Fig. 3:**
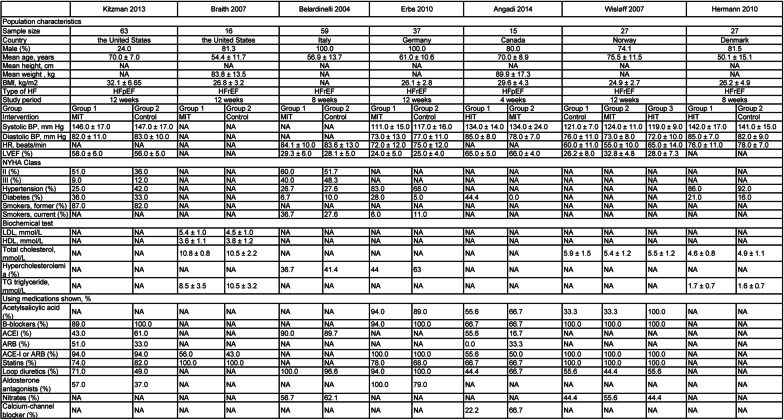
Population characteristics of included studies involving flow-mediated dilation and exercises in patients with chronic heart failure

Figure 2 presents the characteristics of included trials comparing the arterial tonometric technologies FMD and AIx between patients with CHF and healthy participants, while Fig. 3 shows the characteristics of trials referring to FMD in the cases related to exercise.

### Description of randomized controlled trials

The trials comprised 876 participants. Mean age varied from 44.8 to 75.5 years. The proportion of male participants ranged from 24 to 100%. The countries of the studies were the United States (n = 5, 27.8%), Italy (n = 3, 16.7%), Australia (n = 2, 11.1%), Germany (n = 2, 11.1%), Canada (n = 2, 11.1%), Brazil (n = 1, 5.6%), the United Kingdom (n = 1, 5.6%), Norway (n = 1, 5.6%), and Denmark (n = 1, 5.6%). In the studies involving exercise, testing duration ranged from 4 to 12 weeks.

### Quality assessment

We assessed the risk of bias of all the included articles, and in Additional file 1: Fig. S1 shows the risk of bias. Several trials did not mention sufficient details to allow us to assess the risk of bias, especially selection bias, including random sequence generation and allocation concealment, wherein a low risk of bias was mentioned in 10 (55.6%) and 11 (61.1%) articles, respectively. The same proportion of a low risk of bias existed in performance bias and detection bias (n = 15, 83.3%). The other three items were almost not observed in the included studies.

### Outcomes

#### Flow-mediated dilation

**Fig. 4:**
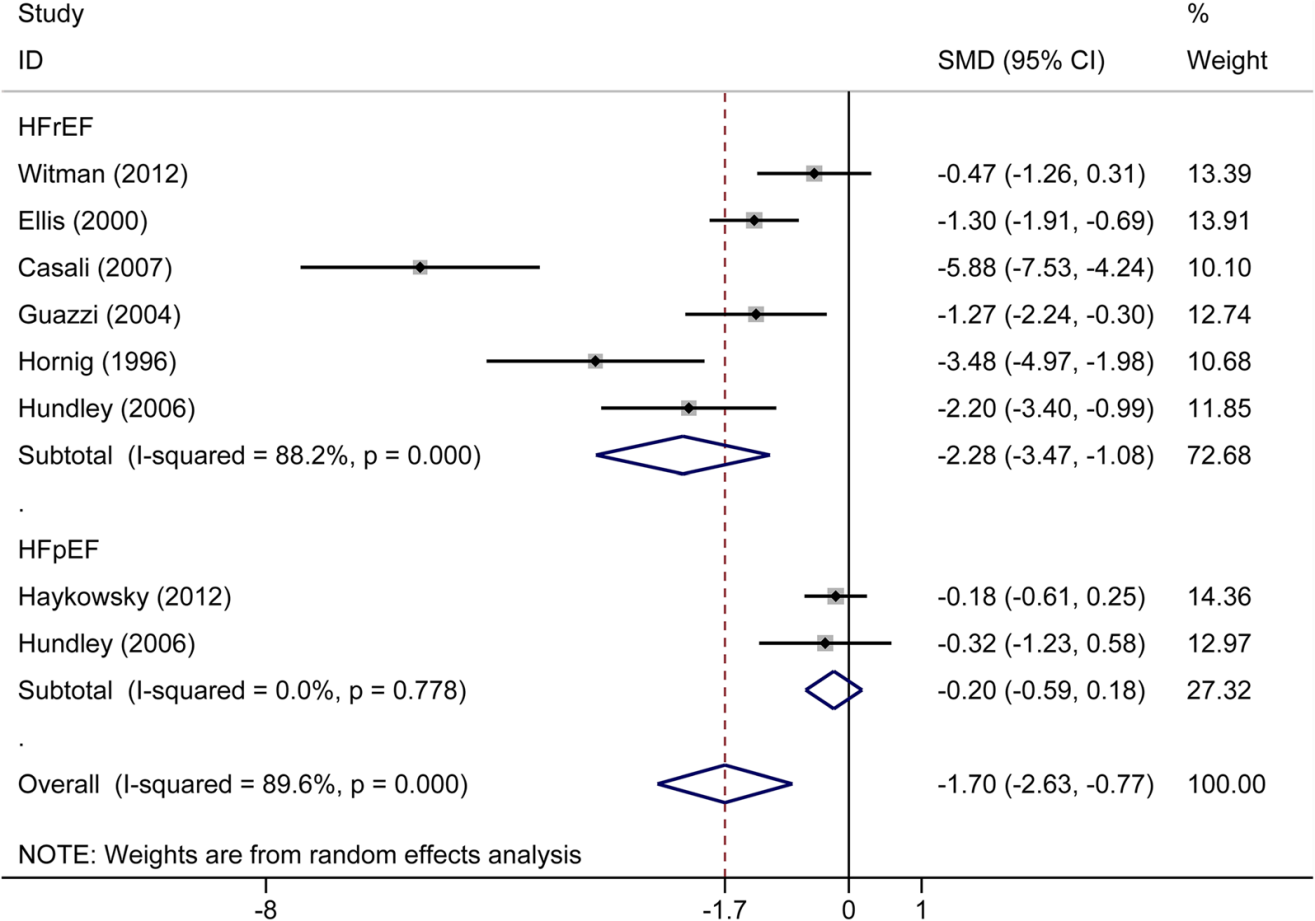
Forest plot of the subgroup analysis of flow-mediated dilation in patients with chronic heart failure and healthy participants based on different types of chronic heart failure. HFrEF, heart failure with reduced ejection fraction; HFpEF, heart failure with preserved ejection fraction

Seven articles containing 301 participants compared the FMD between 190 patients with CHF and 111 healthy subjects. Based on a random-effects model, FMD was significantly lower in patients with CHF (SMD: -1.70%, 95% CI − 2.63 to − 0.77, *P* < 0.001) (Fig. 4). At 13.7% relative risk reduction (RRR), TSA showed that the mean difference (MD) was − 2.63%, and the TSA-adjusted CI was − 3.25 to − 1.96 (*P* < 0.0001). Based on a random-effect model, the blue z-curve crossed trial sequential monitoring (TSM) boundary in the graph above and the green z-curve after penalized tests crossed conventional (CON) boundary in the following graph comparing FMD between patients with CHF and healthy participants (Additional file 1: Fig. S2). In the subgroup analyses, meta-analysis indicated that there were significant differences between HFrEF groups and the control groups (six trials, n = 185, SMD: − 2.28%, 95% CI − 3.47 to − 1.08, *P* < 0.001), while there were no significant differences between HFpEF groups and the control groups (two trials, n = 116, SMD: − 0.20%, 95% CI − 0.59 to 0.18, *P* = 0.299). For further validation, subgroup analyses were performed using TSA as well. As illustrated in Additional file 1: Fig. S3, z-curve trends related to comparing patients with HFrEF and healthy subjects were the same as the trends in Additional file 1: Fig. S2 (MD: − 2.83%, TSA-adjusted CI − 3.42 to − 2.31, *P* < 0.0001). However, z-curves did not cross CON and TSM boundaries in patients with HFpEF compared with healthy participants (Additional file 1: Fig. S4) (MD: − 0.45%, TSA-adjusted CI − 4.14 to 3.24, *P* = 0.33). In conclusion, FMD was significantly higher in healthy population than patients with HFrEF, while in patients with HFpEF, further high-quality studies are needed.

**Fig. 5:**
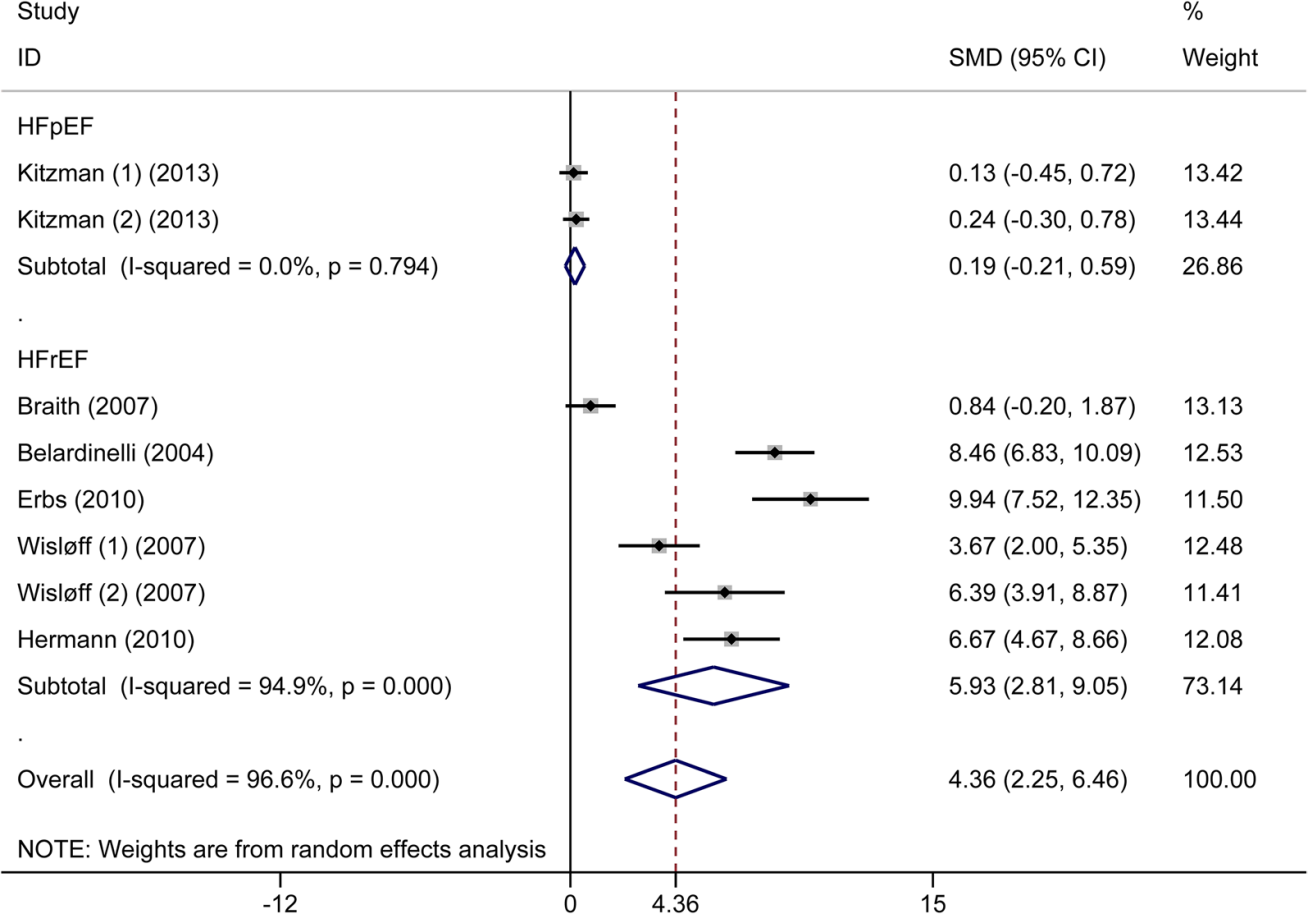
Forest plot of the subgroup analysis of flow-mediated dilation in patients with chronic heart failure in the exercise and non-exercise setting based on different types of chronic heart failure. HFrEF, heart failure with reduced ejection fraction; HFpEF, heart failure with preserved ejection fraction

**Fig. 6:**
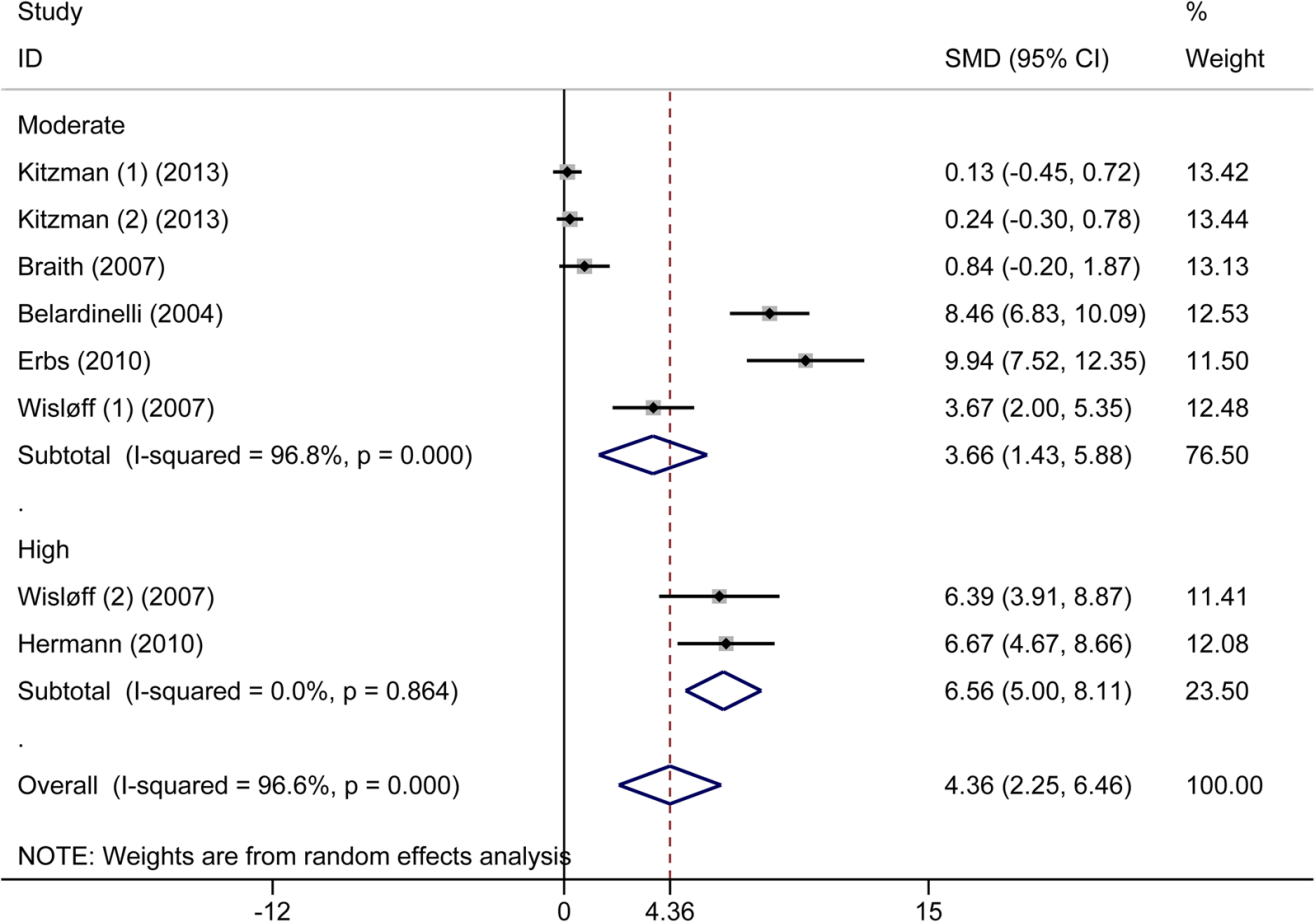
Forest plot of the subgroup analysis of flow-mediated dilation in patients with chronic heart failure in the exercise and non-exercise setting based on different exercise intensities

Six articles, including 270 patients with CHF, compared FMD in the situations of exercise and non-exercise, wherein the overall numbers in both situations were equal. The increases of FMD value were statistically significant in the cases of exercise training (SMD: 4.36%, 95% CI 2.25 to 6.46, *P* < 0.001) based on a random-effects model. At 13.7% RRR, TSA showed that the MD was 3.75, and the TSA-adjusted CI was 1.88 to 5.63 (*P* < 0.0001). Based on a random-effect model, the blue z-curve crossed the TSM boundary in the graph above, and the green z-curve after penalized tests crossed the CON boundary in the following graph comparing FMD between patients with CHF and healthy participants (Additional file 1: Fig. S5). We performed two subgroup analyses using meta-analysis, wherein one was based on types of CHF, and the other was made on exercise intensity. In the first subgroup meta-analysis, there were significant differences in patients with HFrEF after exercise training (six trials, n = 172, SMD: 5.96%, 95%CI 2.81 to 9.05, *P* < 0.001), while there were no significant differences in patients with HFpEF (two trials, n = 98, SMD: 0.19%, 95%CI − 0.21 to 0.59, *P* = 0.348) (Fig. 5). In the second subgroup meta-analysis, there were statistically significant differences after both MIT (five trials, n = 226, SMD: 3.66%, 95%CI 1.43 to 5.88, *P* = 0.001) and HIT (two trials, n = 44, SMD: 6.56%, 95%CI 5.00–8.11, *P* < 0.001) in patients with CHF (Fig. 6). For further verification, we also performed TSA on these two subgroups. For the former subgroup, as showcased in Additional file 1: Figs. S6 and S7, there was statistically significant improvement of FMD in patients with HFrEF after exercise training (MD: 5.04%, TSA-adjusted CI 3.16 to 6.91, *P* < 0.0001), while further high-quality studies are still needed in patients with HFpEF (MD: 0.15%, TSA-adjusted CI − 1.10 to 1.40, *P* = 0.330). For the latter subgroup, as shown in the Additional file 1: Figs. S8 and S9, the blue z-curve crossed the CON boundary, however, after one or two trials the z-curve went below the TSM boundary again, besides, the green z-curve after penalized tests went below the CON boundary again as well. No firm conclusions can be drawn, and further high-quality studies are needed in both MIT(MD: 3.04%, TSA-adjusted CI − 0.18 to 6.26, *P* = 0.006) and HIT (MD: 5.9%, TSA-adjusted CI − 5.29 to 17.12, *P* = 0.021) exercise. In conclusion, FMD significantly improved after exercise training in patients with HFrEF. However, although we got the negative result that there were no significant differences after exercise training in patients with HFpEF and positive results that MIT and HIT were beneficial to the improvement of FMD in patients with CHF, TSA did not further identify these outcomes. Further high-quality studies are needed.

**Fig. 7:**
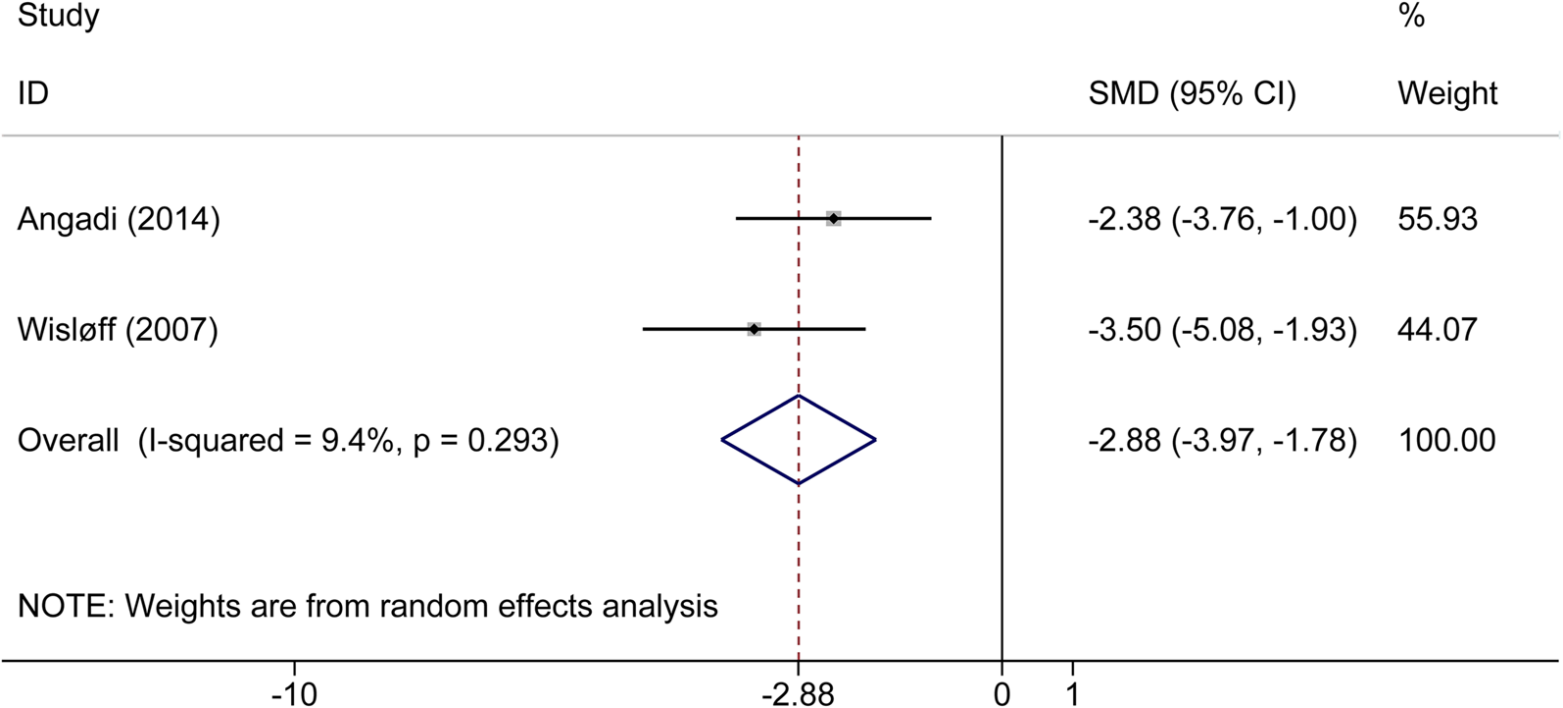
Forest plot of flow-mediated dilation in patients with chronic heart failure in moderate intensity training exercise and high intensity training exercise setting

There were two articles that compared 32 patients with CHF between MIT and HIT exercises. Based on a random-effects model, the improvement of FMD was significantly better in patients with CHF after HIT exercise compared with MIT (SMD: 2.88%, 95% CI 1.78 to 3.97, *P* < 0.001) (Fig. 7). At 13.7% RRR, MD was 3.68%, and the 95% CI was 2.23 to 5.13 (*P* < 0.0001). Based on a random-effect model, the blue z-curve crossed the TSM boundary in the graph above, and the green z-curve after penalized tests crossed the CON boundary in the following graph (Additional file 1: Fig. S10). In conclusion, the improvement of FMD was significantly better in patients with CHF after HIT exercise compared with the patients with CHF after MIT exercise.

#### Augmentation index

**Fig. 8:**
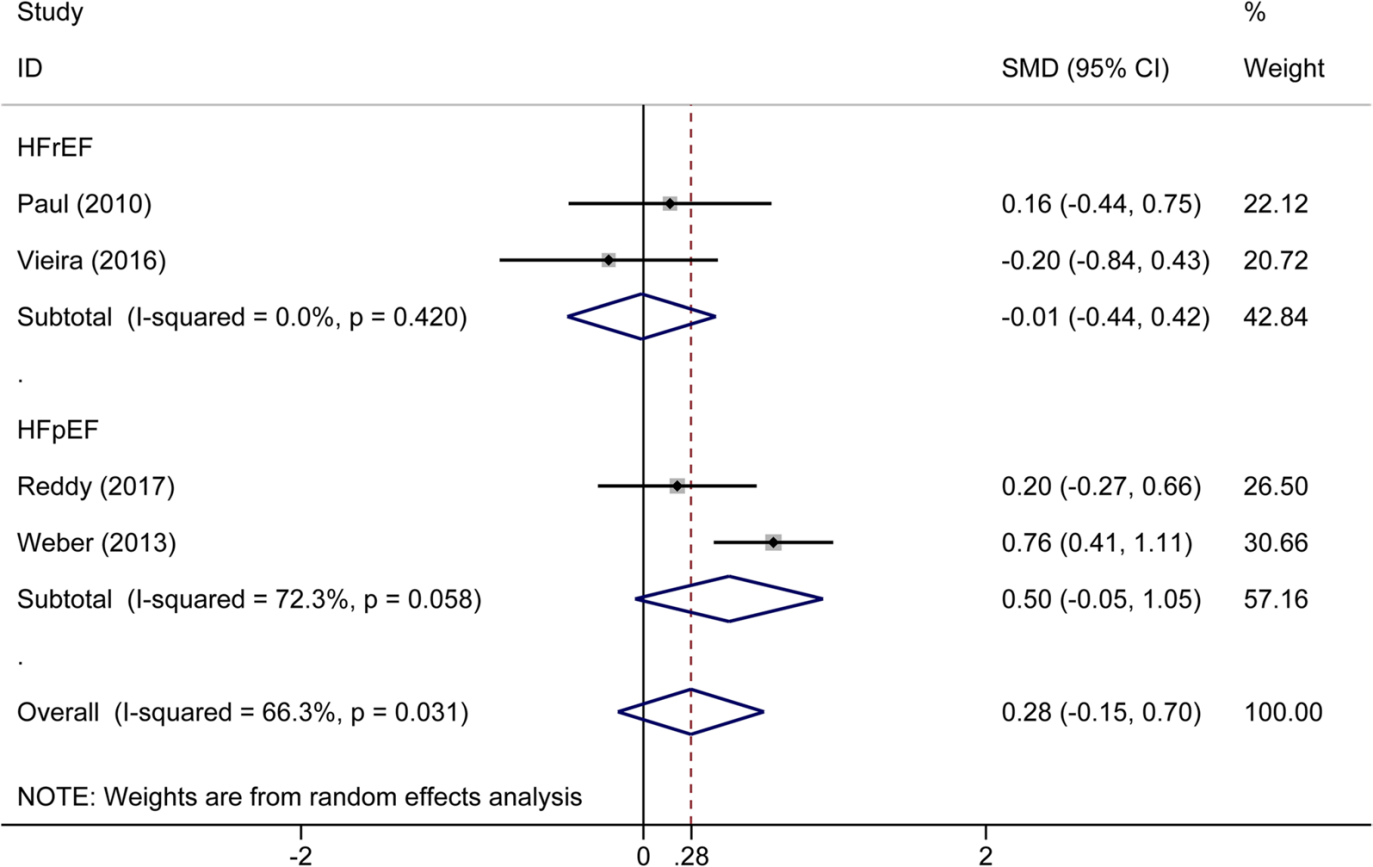
Forest plot of the subgroup analysis of augmentation index in patients with chronic heart failure and healthy participants based on different types of chronic heart failure. HFrEF, heart failure with reduced ejection fraction; HFpEF, heart failure with preserved ejection fraction

Six articles containing 341 patients compared the AIx between 217 patients with CHF and 124 healthy subjects. Based on a random-effects model, AIx was non-significant difference between patients with CHF and healthy subjects (SMD: 0.28%, 95% CI − 0.15 to 0.70, *P* = 0.203) (Fig. 8). At 13.7% RRR, TSA showed that the MD was 3.60% and the TSA-adjusted CI was − 3.39 to 10.57 (*P* = 0.131), and both the blue z-curve in the graph above and the green z-curve after penalized tests in the following graph did not cross the CON boundary based on a random-effect model (Additional file 1: Fig. S11). Further high-quality studies are needed. In the subgroup meta-analyses, there were no statistically significant differences both in patients with HFrEF (two trials, n = 85, SMD: − 0.01%, 95% CI − 0.44 to 0.42, *P* = 0.961) and HFpEF (two trials, n = 256, SMD: 0.50%, 95% CI − 0.05 to 1.05, *P* = 0.074) compared with the control groups. For further validation, subgroup analyses were also performed using TSA on HFpEF and HFrEF. As illustrated in Additional file 1: Figs. S12 and S13, the blue z-curve crossed the TSM boundary, and the green z-curve after penalized tests crossed the CON boundary in patients with HFpEF (MD: 6.56%, TSA-adjusted CI 0.30 to 12.82, *P* = 0.004), while z-curves did not cross the CON or TSM boundaries in patients with HFrEF (MD: 0.68%, 95% CI − 3.66 to 5.01, *P* = 0.760) and further high-quality studies are needed. In conclusion, there were no significant differences in AIx in patients with HFpEF, while more high-quality studies are needed to further test whether there are significant differences in AIX in patients with HFrEF compared with healthy population.

### Sensitivity analysis

Sensitivity analyses indicated that the pooled outcomes of FMD and AIx between patients with CHF and healthy participants and FMD between patients with CHF in the cases of exercise and non-exercise did not change significantly when each study was excluded one by one (Additional file 1: Figs. S14 to S16). The sensitivity analyses did not influence the heterogeneity of FMD and AIx in these situations.

### Meta-regression analysis

As illustrated in Table 1, there was a significantly higher proportion of males in evaluating FMD in patients with CHF between exercise and non-exercise (*P* = 0.015). For the rest, there was no evidence of a correlation between the change of the two indexes and any other covariates in univariate meta-regression analyses.

**Table 1:** Univariate meta-regression analyses

	*P* values		
	AIx in patients with CHF and healthy subjects	FMD in patients with CHF in the cases of exercise and non-exercise	FMD in patients with CHF and healthy subjects
Sample size	0.202	0.929	0.457
Publication date	0.684	0.212	0.375
Type of CHF	0.293	0.059	0.105
Exercise intensity	–	0.343	–
Male (%)	0.404	0.015	0.182
Mean age, years	0.453	0.471	0.214
Overall risk of bias	0.249	0.331	0.247
Random sequence generation	0.313	0.238	0.149
Allocation concealment	0.330	0.524	0.157
Blinding of participants and personnel	0.276	0.931	0.238
Blinding of outcome assessment	0.234	0.090	0.229

AIx, augmentation index; CHF, chronic heart failure; FMD, flow-mediated dilation

## Discussion

This meta-analysis sought to find correlations between exercise and common arterial tonometric technologies deeply involved with arterial stiffness in patients with CHF. In the final view, there were 20 articles considered. However, considering that only two studies involved pulse wave velocity and one study involved AIx@75, there was insufficient evidence to conduct a meta-analysis. Therefore, we performed this study based on FMD and AIx, and 18 articles were finally included. FMD is measured through the brachial artery diameter before and after cuff occlusion by ultrasound which simulates the response of arterial to exercise, and this index is commonly acknowledged to be a standardized noninvasive method to assess arterial stiffness [60–62]. AIx is measured as the augmentation of aortic systolic blood pressure through the reflected pulse wave and presented by a percentage of the aortic pulse pressure and is also regarded as an effective marker of arterial stiffness [63–65].

Reviewing the 18 included articles, we compared these two indexes between patients with CHF and healthy subjects and compared the changes of FMD in patients with CHF after different intensity exercises.

For the index FMD, the primary finding showed that this index was significantly lower in patients with CHF compared with healthy subjects. In subgroup meta-analysis, the outcome indicated that this index was significantly lower in patients with HFrEF compared with healthy participants, while there were no significant distinctions between the HFpEF subgroup and the control group. The TSA showed that the research outcome about the HFrEF subgroup was further verified, while the number of the HFpEF subgroup was too small to reach a definitive conclusion, and TSA calculated the optimum sample size. Second, we found that FMD improved significantly in patients with CHF after exercise training compared with non-exercise training. We performed this point using meta-analysis by two subgroups. In one subgroup, meta-analysis was conducted based on types of CHF, the finding showed similar results to the outcomes above, wherein FMD improved significantly in the HFrEF subgroup while not in the HFpEF subgroup. Similarly, TSA further confirmed the outcome of the HFrEF subgroup, while it did not provide a definite conclusion in the HFpEF subgroup, and further high-quality studies are needed to look into that. In another subgroup meta-analysis based on exercise intensity, FMD improved significantly in patients with CHF after both MIT and HIT exercise compared with non-exercise. However, TSA indicated that the number of these two subgroups was not enough to obtain definite results, and more high-quality studies are needed. Third, the improvement of FMD was more remarkable after HIT exercise in patients with CHF compared with MIT exercise, and this outcome was further confirmed by TSA.

For another index AIx, the outcome indicated no significant differences between the patients with CHF and healthy participants. In the subgroup meta-analysis, we also did not find any significant distinctions in both HFrEF and HFpEF groups compared with the control group. To our surprise, the outcome that there was no significant difference of AIx between patients with HFpEF and healthy participants was further proved by TSA, while in the HFrEF subgroup, more high-quality studies are needed to further confirm the outcome in the future.

Obvious statistical heterogeneity existed in some of the pooled comparisons. There may be related clinical and methodological distinctions in the studies. Some distinctions existed in the inclusion criteria and within the subjects, which differed in gender ratio and underlying diseases, came from different countries, and took different medicines when participating in the studies. The testing duration and testing form also varied among the studies.

### Strengths and limitations

As far as we know, this article is the first meta-analysis used in combination with TSA to assess the relationship between arterial tonometry and exercise in patients with CHF based on RCT. Unlike a prior published meta-analysis, it was performed through single-arm meta-analysis and compared arterial tonometry with echocardiographic indexes of cardiac diastolic dysfunction [66]. We only adopted RCT in the final analysis to sustain consistency and secure potent pooled estimates, and there are also obvious differences in the research content between the prior meta-analysis and ours. Besides, we combined TSA to control the type I and type II errors of conventional meta-analysis better.

Our meta-analysis has several limitations. First, despite all the included studies being RCTs, only 10 (55.6%) articles indicated random sequence generation, and 11 (61.1%) articles indicated allocation concealment. Accordingly, selection bias may be present. Second, women are more susceptible to arterial stiffness[67], while in the 14 (77.8%) included articles, more than half of the subjects were male, and even in 4 (22.2%) articles, all the participants were male. Such a situation may lead to the pooled estimates we got being underrated. Third, as of the search date, only a small number of related articles were available that led to wide 95%CI for point estimates in this meta-analysis and therefore limited the power of our research results. More high-quality RCTs are needed in the future. Forth, although we systematically search MEDLINE, EMBASE, and Cochrane Central Register of Controlled Trials (CENTRAL) in The Cochrane Library for related articles by using a comprehensive search strategy and we also reviewed the reference lists of eligible studies and systematic reviews to search the relevant articles, we did not retrieve any unpublished studies. Therefore, the potential publication bias may occur in our meta-analysis.

## Conclusions

Our study showed that FMD was lower in basal condition and improved significantly after exercise in patients with HFrEF compared with healthy participants. HIT exercise was more beneficial to improve FMD in patients with CHF compared with MIT exercise. For AIx, our study indicated no significant differences in patients with HFpEF compared with healthy participants.

In addition, we got negative results that there are no significant differences of FMD in basal condition and after exercise in patients with HFpEF compared with healthy participants, and there were no significant differences of AIx in patients with HFrEF compared with healthy participants. Besides, we also got positive results that there were statistically significant differences after both MIT exercise and HIT exercise in patients with CHF. However, these outcomes were not identified after further verification using TSA, and more high-quality studies are needed to reach definitive conclusions in the future.

## Supplementary Information

**Additional file 1: Fig. S1.** Risk of bias of all included articles. **Fig. S2.** Trial sequential analysis for flow-mediated dilation between patients with chronic heart failure and healthy participants. **Fig. S3.** Trial sequential analysis for flow-mediated dilation between patients with heart failure with reduced ejection fraction and healthy participants. **Fig. S4.** Trial sequential analysis for flow-mediated dilation between patients with heart failure with preserved ejection fraction and healthy participants. **Fig. S5.** Trial sequential analysis for flow-mediated dilation in patients with chronic heart failure in the cases of exercise and non-exercise. **Fig. S6.** Trial sequential analysis for flow-mediated dilation in patients with heart failure with reduced ejection fraction in the cases of exercise and non-exercise. **Fig. S7.** Trial sequential analysis for flow-mediated dilation in patients with heart failure with preserved ejection fraction in the cases of exercise and non-exercise. **Fig. S8.** Trial sequential analysis for flow-mediated dilation in patients with chronic heart failure in the cases of moderate-intensity exercise training and non-exercise training. **Fig. S9.** Trial sequential analysis for flow-mediated dilation in patients with chronic heart failure in the cases of high-intensity exercise training and non-exercise training. **Fig. S10.** Trial sequential analysis for flow-mediated dilation in patients with chronic heart failure in the cases of high-intensity exercise training and moderate-intensity exercise training. **Fig. S11.** Trial sequential analysis for augmentation Index between patients with chronic heart failure and healthy participants. **Fig. S12.** Trial sequential analysis for augmentation Index between patients with heart failure with preserved ejection fraction and healthy participants. **Fig. S13.** Trial sequential analysis for augmentation Index between patients with heart failure with reduced ejection fraction and healthy participants. **Fig. S14.** Sensitivity analysis of flow-mediated dilation in patients with chronic heart failure and healthy participants. **Fig. S15.** Sensitivity analysis of augmentation index in patients with chronic heart failure and healthy participants. **Fig. S16.** Sensitivity analysis of flow-mediated dilation in patients with chronic heart failure in the cases of exercise and non-exercise.

## Data Availability

The datasets used and analysed during the current study are available from the correspondence author.

## Abbreviations

CHF: Chronic heart failure
TSA: Trial sequential analysis
FMD: Flow-mediated dilation
SMD: Standardized mean difference
CI: Confidence interval
HFrEF: Heart failure with reduced ejection fraction
HFpEF: Heart failure with preserved ejection fraction
AIx: Augmentation index
MIT: Moderate-intensity training
HIT: High-intensity training
RCT: Randomized controlled trials
IQR: Interquartile range
SD: Standard deviation
SE: Standard error
RIS: Required information size
RRR: Relative risk reduction
MD: Mean difference
TSM: Trial sequential monitoring
CON: Conventional
