# Supplementary material for: Relationship of arterial tonometry and exercise in patients with chronic heart failure: a systematic review with meta-analysis and trial sequential analysis
Source: BMC Cardiovasc Disord. 2022 Aug 1;22:345. doi: 10.1186/s12872-022-02792-6 (PMC9341099; doi:10.1186/s12872-022-02792-6)

**Relationship of arterial tonometry and exercise in patients with chronic heart failure: a systematic review with meta-analysis and trial sequential analysis - eSupplement**

**1. Search strategy**

**I.** Search Strategy for MEDLINE.

#1 "Vascular Stiffness"[Mesh] 7,523

#2 (((((((Stiffness, Vascular[Title/Abstract]) OR (Vascular Stiffnesses[Title/Abstract])) OR (Arterial Stiffness[Title/Abstract])) OR (Arterial Stiffnesses[Title/Abstract])) OR (Stiffness, Arterial[Title/Abstract])) OR (Aortic Stiffness[Title/Abstract])) OR (Aortic Stiffnesses[Title/Abstract])) OR (Stiffness, Aortic[Title/Abstract]) 12,424

#3 #1 OR #2 14,125

#4 "Pulse Wave Analysis"[Mesh] 5,537

#5 ((((((((((((((((((Analyses, Pulse Wave[Title/Abstract]) OR (Analysis, Pulse Wave[Title/Abstract])) OR (Pulse Wave Analyses[Title/Abstract])) OR (Wave Analyses, Pulse[Title/Abstract])) OR (Wave Analysis, Pulse[Title/Abstract])) OR (Pulse Wave Velocity[Title/Abstract])) OR (Pulse Wave Velocities[Title/Abstract])) OR (Velocities, Pulse Wave[Title/Abstract])) OR (Velocity, Pulse Wave[Title/Abstract])) OR (Wave Velocities, Pulse[Title/Abstract])) OR (Wave Velocity, Pulse[Title/Abstract])) OR (Pulse Transit Time[Title/Abstract])) OR (Pulse Transit Times[Title/Abstract])) OR (Time, Pulse Transit[Title/Abstract])) OR (Times, Pulse Transit[Title/Abstract])) OR (Transit Time, Pulse[Title/Abstract])) OR (Transit Times, Pulse[Title/Abstract])) OR (Pulse Wave Transit Time[Title/Abstract])) OR (PWV[Title/Abstract]) 11,992

#6 #4 OR #5 13,020

#7 ((augmentation index[Title/Abstract]) OR (AI[Title/Abstract])) OR (AIx[Title/Abstract]) 44,758

#8 ((arterial pressure volume index[Title/Abstract]) OR (arterial pressure index[Title/Abstract]) ) OR (API[Title/Abstract]) 10,027

#9 ((arterial velocity pulse index[Title/Abstract]) OR (arterial velocity index[Title/Abstract]) ) OR (AVI[Title/Abstract]) 877

#10 ((cardio-ankle vascular index[Title/Abstract]) OR (CAVI[Title/Abstract]) ) OR (cardio ankle vascular index[Title/Abstract]) 882

#11 "Ankle Brachial Index"[Mesh] 3,711

#12 (((((((((((((((((((Ankle Brachial Indices[Title/Abstract]) OR (Brachial Index, Ankle[Title/Abstract])) OR (Brachial Indices, Ankle[Title/Abstract])) OR (Index, Ankle Brachial[Title/Abstract])) OR (Indices, Ankle Brachial[Title/Abstract])) OR (Ankle-Brachial Index[Title/Abstract])) OR (Ankle-Brachial Indices[Title/Abstract])) OR (Index, Ankle-Brachial[Title/Abstract])) OR (Indices, Ankle-Brachial[Title/Abstract])) OR (Toe Brachial Index[Title/Abstract])) OR (Brachial Index, Toe[Title/Abstract])) OR (Brachial Indices, Toe[Title/Abstract])) OR (Index, Toe Brachial[Title/Abstract])) OR (Indices, Toe Brachial[Title/Abstract])) OR (Toe Brachial Indices[Title/Abstract])) OR (Toe-Brachial Index[Title/Abstract])) OR (Index, Toe-Brachial[Title/Abstract])) OR (Indices, Toe-Brachial[Title/Abstract])) OR (Toe-Brachial Indices[Title/Abstract])) OR (ABI[Title/Abstract]) 10,261

#13 #11 OR #12 11,685

#14 ((flow-mediated dilation[Title/Abstract]) OR (FMD[Title/Abstract]) ) OR (flow mediated dilation[Title/Abstract]) 9,912

#15 #3 OR #6 OR #7 OR #8 OR #9 OR #10 OR #13 OR #14 91,578

#16 "Heart Failure"[Mesh] 135,747

#17 (((((((((((((Cardiac Failure[Title/Abstract]) OR (Heart Decompensation[Title/Abstract])) OR (Decompensation, Heart[Title/Abstract])) OR (Heart Failure, Right-Sided[Title/Abstract])) OR (Heart Failure, Right Sided[Title/Abstract])) OR (Right-Sided Heart Failure[Title/Abstract])) OR (Right Sided Heart Failure[Title/Abstract])) OR (Myocardial Failure[Title/Abstract])) OR (Congestive Heart Failure[Title/Abstract])) OR (Heart Failure, Congestive[Title/Abstract])) OR (Heart Failure, Left-Sided[Title/Abstract])) OR (Heart Failure, Left Sided[Title/Abstract])) OR (Left-Sided Heart Failure[Title/Abstract])) OR (Left Sided Heart Failure[Title/Abstract]) 57,826

#18 #16 OR #17 166,010

#19 randomized controlled trial[Publication Type] OR randomized[Title/Abstract] OR placebo[Title/Abstract] 938,451

#20 #15 AND #18 AND #19 119

**II. Search Strategy for EMBASE.**

#1. 'arterial stiffness'/exp 25,042

#2. 'stiffness, vascular':ab,ti 81

#3. 'vascular stiffnesses':ab,ti 1

#4. 'arterial stiffness':ab,ti 18,514

#5. 'arterial stiffnesses':ab,ti 4

#6. 'stiffness, arterial':ab,ti 121

#7. 'aortic stiffness':ab,ti 18,514

#8. 'aortic stiffnesses':ab,ti 2

#9. 'stiffness, aortic':ab,ti 187

#10. #1 OR #2 OR #3 OR #4 OR #5 OR #6 OR #7 OR #8 OR #9 27,996

#11. 'pulse wave velocity'/exp 3,152

#12. 'analyses, pulse wave':ab,ti 5

#13. 'analysis, pulse wave':ab,ti 28

#14. 'pulse wave analyses':ab,ti 37

#15. 'wave analyses, pulse':ab,ti 0

#16. 'wave analysis, pulse':ab,ti 23

#17. 'pulse wave velocity':ab,ti 19,306

#18. 'pulse wave analysis':ab,ti 1,879

#19. 'pulse wave velocities':ab,ti 235

#20. 'velocities, pulse wave':ab,ti 1

#21. 'velocity, pulse wave':ab,ti 25

#22. 'wave velocities, pulse':ab,ti 3

#23. 'wave velocity, pulse':ab,ti 45

#24. 'pulse transit time':ab,ti 889

#25. 'pulse transit times':ab,ti 31

#26. 'time, pulse transit':ab,ti 3

#27. 'times, pulse transit':ab,ti 0

#28. 'transit time, pulse':ab,ti 10

#29. 'transit times, pulse':ab,ti 0

#30. 'pulse wave transit time':ab,ti 145

#31. 'pwv':ab,ti 11,963

#32. #11 OR #12 OR #13 OR #14 OR #15 OR #16 OR #17 OR

#18 OR #19 OR #20 OR #21 OR #22 OR #23 OR #24 OR

#25 OR #26 OR #27 OR #28 OR #29 OR #30 OR #31 21,959

#33. 'augmentation index':ab,ti 6,472

#34. 'aix':ab,ti 3,498

#35. 'ai':ab,ti 55,073

#36. #33 OR #34 OR #35 61,063

#37. 'arterial pressure volume index':ab,ti 24

#38. 'arterial pressure index':ab,ti 46

#39. 'api':ab,ti 14,396

#40. #37 OR #38 OR #39 14,433

#41. 'arterial velocity pulse index':ab,ti 35

#42. 'arterial velocity index':ab,ti 1

#43. 'avi':ab,ti 1,301

#44. #41 OR #42 OR #43 1,302

#45. 'cardio-ankle vascular index':ab,ti 1,221

#46. 'cavi':ab,ti 1,457

#47. #45 OR #46 1,599

#48. 'ankle brachial index'/exp 12,357

#49. 'ankle brachial indices':ab,ti 344

#50. 'brachial index, ankle':ab,ti 3

#51. 'brachial indices, ankle':ab,ti 0

#52. 'index, ankle brachial':ab,ti 49

#53. 'indices, ankle brachial':ab,ti 2

#54. 'ankle-brachial index':ab,ti 7,988

#55. 'ankle-brachial indices':ab,ti 345

#56. 'index, ankle-brachial':ab,ti 49

#57. 'indices, ankle-brachial':ab,ti 2

#58. 'toe brachial index':ab,ti 311

#59. 'brachial index, toe':ab,ti 40

#60. 'brachial indices, toe':ab,ti 5

#61. 'index, toe brachial':ab,ti 40

#62. 'indices, toe brachial':ab,ti 0

#63. 'toe brachial indices':ab,ti 31

#64. 'toe-brachial index':ab,ti 311

#65. 'index, toe-brachial':ab,ti 40

#66. 'indices, toe-brachial':ab,ti 0

#67. 'toe-brachial indices':ab,ti 31

#68. #48 OR #49 OR #50 OR #51 OR #52 OR #53 OR #54 OR

#55 OR #56 OR #57 OR #58 OR #59 OR #60 OR #61 OR

#62 OR #63 OR #64 OR #65 OR #66 OR #67 13,871

#69. 'heart failure'/exp 588,788

#70. 'cardiac failure':ab,ti 18,315

#71. 'heart decompensation':ab,ti 153

#72. 'decompensation, heart':ab,ti 19

#73. 'heart failure, right-sided':ab,ti 5

#74. 'heart failure, right sided':ab,ti 5

#75. 'right sided heart failure':ab,ti 1,144

#76. 'right sided heart failure':ab,ti 1,144

#77. 'myocardial failure':ab,ti 954

#78. 'congestive heart failure':ab,ti 57,241

#79. 'heart failure, congestive':ab,ti 61

#80. 'heart failure, left-sided':ab,ti 4

#81. 'heart failure, left sided':ab,ti 4

#82. 'left-sided heart failure':ab,ti 388

#83. 'left sided heart failure':ab,ti 388

#84. #69 OR #70 OR #71 OR #72 OR #73 OR #74 OR #75 OR

#76 OR #77 OR #78 OR #79 OR #80 OR #81 OR #82 OR

#83 605,968

#85. 'random':ab,ti OR 'placebo':ab,ti OR 'double-blind':ab,ti 773,721

#86. #10 OR #32 OR #36 OR #40 OR #44 OR #47 OR #68 119,141

#87. #84 AND #85 AND #86 164

**III. Search Strategy for** **the Cochrane Library.**

#1. MeSH descriptor: [Vascular Stiffness] explode all trees 649

#2. (Stiffness, Vascular):ti,ab,kw OR (Vascular Stiffnesses):ti,ab,kw OR (Arterial Stiffness):ti,ab,kw OR (Arterial Stiffnesses):ti,ab,kw OR (Stiffness, Arterial):ti,ab,kw (Word variations have been searched) 3,651

#3. (Aortic Stiffness):ti,ab,kw OR (Aortic Stiffnesses):ti,ab,kw OR (Stiffness, Aortic):ti,ab,kw (Word variations have been searched) 900

#4. #1 OR #2 OR #3 3,709

#5. MeSH descriptor: [Pulse Wave Analysis] explode all trees 432

#6. (Analyses, Pulse Wave):ti,ab,kw OR (Analysis, Pulse Wave):ti,ab,kw OR (Pulse Wave Analyses):ti,ab,kw OR (Wave Analyses, Pulse):ti,ab,kw OR (Wave Analysis, Pulse):ti,ab,kw (Word variations have been searched) 2,067

#7. (Pulse Wave Velocity):ti,ab,kw OR (Pulse Wave Velocities):ti,ab,kw OR (Velocities, Pulse Wave):ti,ab,kw OR (Velocity, Pulse Wave):ti,ab,kw OR (Wave Velocities, Pulse):ti,ab,kw (Word variations have been searched) 2,871

#8. (Wave Velocity, Pulse):ti,ab,kw OR (Pulse Transit Time):ti,ab,kw OR (Pulse Transit Times):ti,ab,kw OR (Time, Pulse Transit):ti,ab,kw OR (Times, Pulse Transit):ti,ab,kw (Word variations have been searched) 3,032

#9. (Transit Time, Pulse):ti,ab,kw OR (Transit Times, Pulse):ti,ab,kw OR (Pulse Wave Transit Time):ti,ab,kw OR (PWV):ti,ab,kw (Word variations have been searched) 1,930

#10. #5 OR #6 OR #7 OR #8 OR #9 3,944

#11. (augmentation index):ti,ab,kw OR (AIx):ti,ab,kw OR (AI):ti,ab,kw (Word variations have been searched) 11,361

#12. (arterial pressure volume index):ti,ab,kw OR (arterial pressure index):ti,ab,kw OR (API):ti,ab,kw (Word variations have been searched) 13,140

#13. (arterial velocity pulse index):ti,ab,kw OR (arterial velocity index):ti,ab,kw OR (AVI):ti,ab,kw (Word variations have been searched) 3,127

#14. (cardio-ankle vascular index):ti,ab,kw OR (cardio ankle vascular index):ti,ab,kw OR (CAVI):ti,ab,kw (Word variations have been searched) 280

#15. MeSH descriptor: [Ankle Brachial Index] explode all trees 233

#16. (ABI):ti,ab,kw OR (Ankle Brachial Indices):ti,ab,kw OR (Brachial Index, Ankle):ti,ab,kw OR (Brachial Indices, Ankle):ti,ab,kw OR (Index, Ankle Brachial):ti,ab,kw (Word variations have been searched) 2,533

#17. (Indices, Ankle Brachial):ti,ab,kw OR (Ankle-Brachial Index):ti,ab,kw OR (Ankle-Brachial Indices):ti,ab,kw OR (Index, Ankle-Brachial):ti,ab,kw OR (Indices, Ankle-Brachial):ti,ab,kw (Word variations have been searched) 1,721

#18. (Toe Brachial Index):ti,ab,kw OR (Brachial Index, Toe):ti,ab,kw OR (Brachial Indices, Toe):ti,ab,kw OR (Index, Toe Brachial):ti,ab,kw OR (Indices, Toe Brachial):ti,ab,kw (Word variations have been searched) 143

#19. (Toe Brachial Indices):ti,ab,kw OR (Toe-Brachial Index):ti,ab,kw OR (Index, Toe-Brachial):ti,ab,kw OR (Indices, Toe-Brachial):ti,ab,kw OR (Toe-Brachial Indices):ti,ab,kw (Word variations have been searched) 143

#20. #15 OR #16 OR #17 OR #18 OR #19 2,549

#21. (flow-mediated dilation):ti,ab,kw OR (flow mediated dilation):ti,ab,kw OR (FMD):ti,ab,kw (Word variations have been searched) 3,672

#22. #4 OR #10 OR #11 OR #12 OR #13 OR #14 OR #20 OR #21 32,312

#23. MeSH descriptor: [Heart Failure] explode all trees 10,290

#24. (Cardiac Failure):ti,ab,kw OR (Heart Decompensation):ti,ab,kw OR (Decompensation, Heart):ti,ab,kw OR (Heart Failure, Right-Sided):ti,ab,kw OR (Heart Failure, Right Sided):ti,ab,kw (Word variations have been searched) 18,260

#25. (Right-Sided Heart Failure):ti,ab,kw OR (Right Sided Heart Failure):ti,ab,kw OR (Myocardial Failure):ti,ab,kw OR (Congestive Heart Failure):ti,ab,kw OR (Heart Failure, Congestive):ti,ab,kw (Word variations have been searched) 16,265

#26. (Heart Failure, Left-Sided):ti,ab,kw OR (Heart Failure, Left Sided):ti,ab,kw OR (Left-Sided Heart Failure):ti,ab,kw OR (Left Sided Heart Failure):ti,ab,kw (Word variations have been searched) 1,341

#27. #23 OR #24 OR #25 OR #26 31,359

#28. #22 AND #27 1,855

**Fig. S1.** Risk of bias of all included articles.


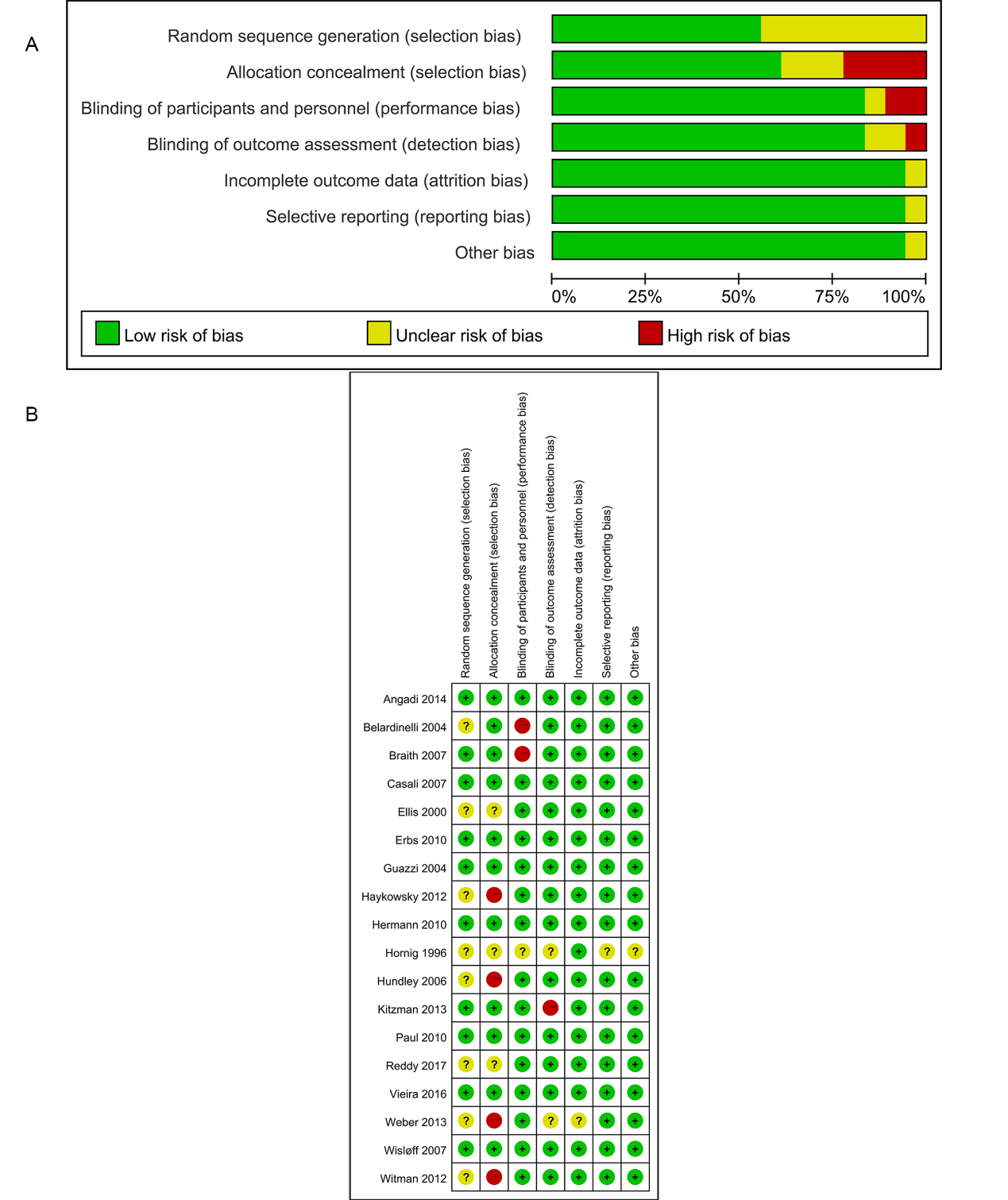


**Fig. S2.** Trial sequential analysis for flow-mediated dilation between patients with chronic heart failure and healthy participants.


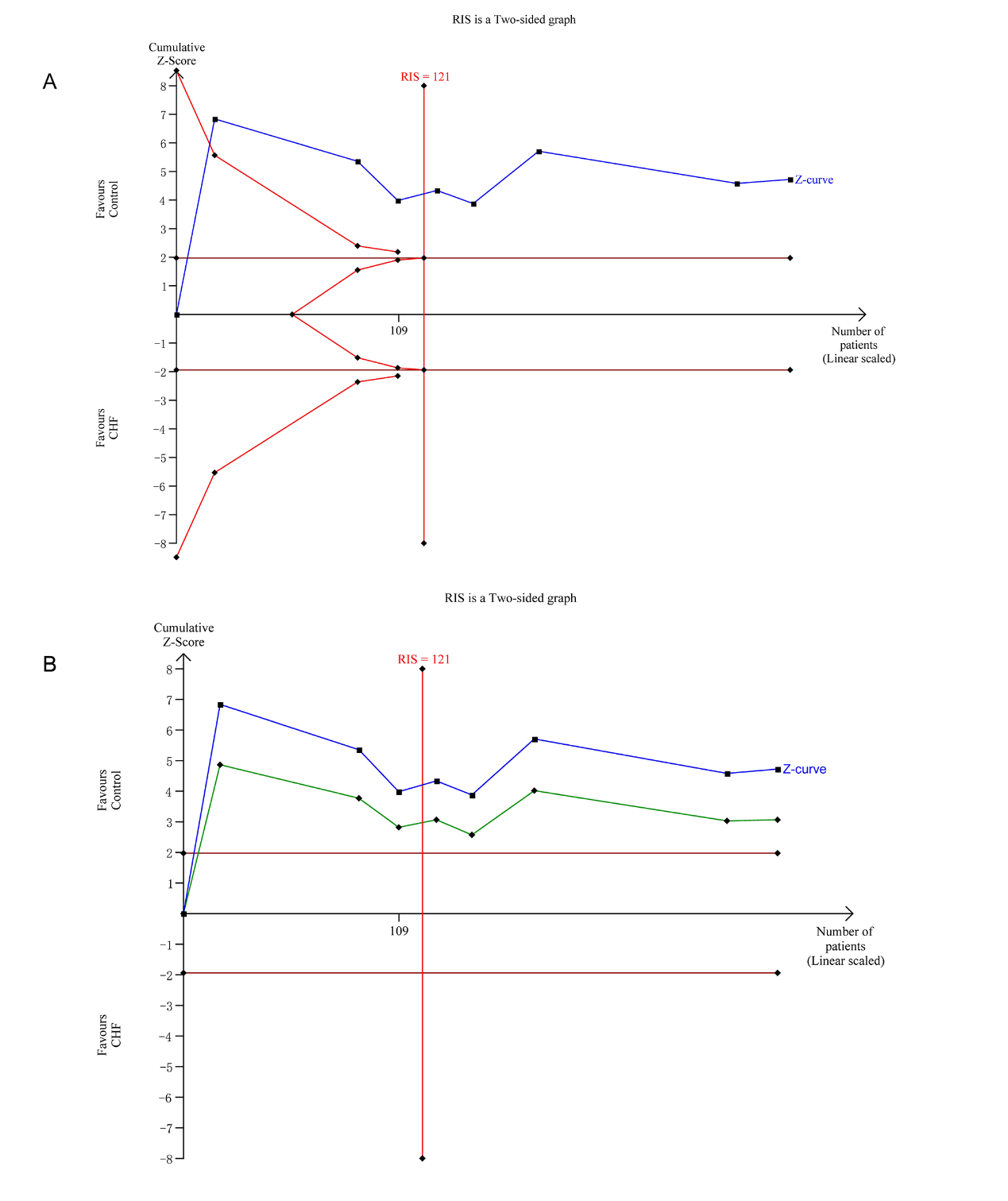


An estimated required information size (RIS) of 121 patients was calculated based on an anticipated relative risk reduction (RRR) of 13.7%, α=0.05 (two-sided), β=0.20 (power=80%). Based on a random-effect model, the blue z-curve crossed trial sequential monitoring boundary in the graph above (A) and the green z-curve after penalized tests crossed conventional boundary in the following graph (B).

**Fig. S3.** Trial sequential analysis for flow-mediated dilation between patients with heart failure with reduced ejection fraction and healthy participants.


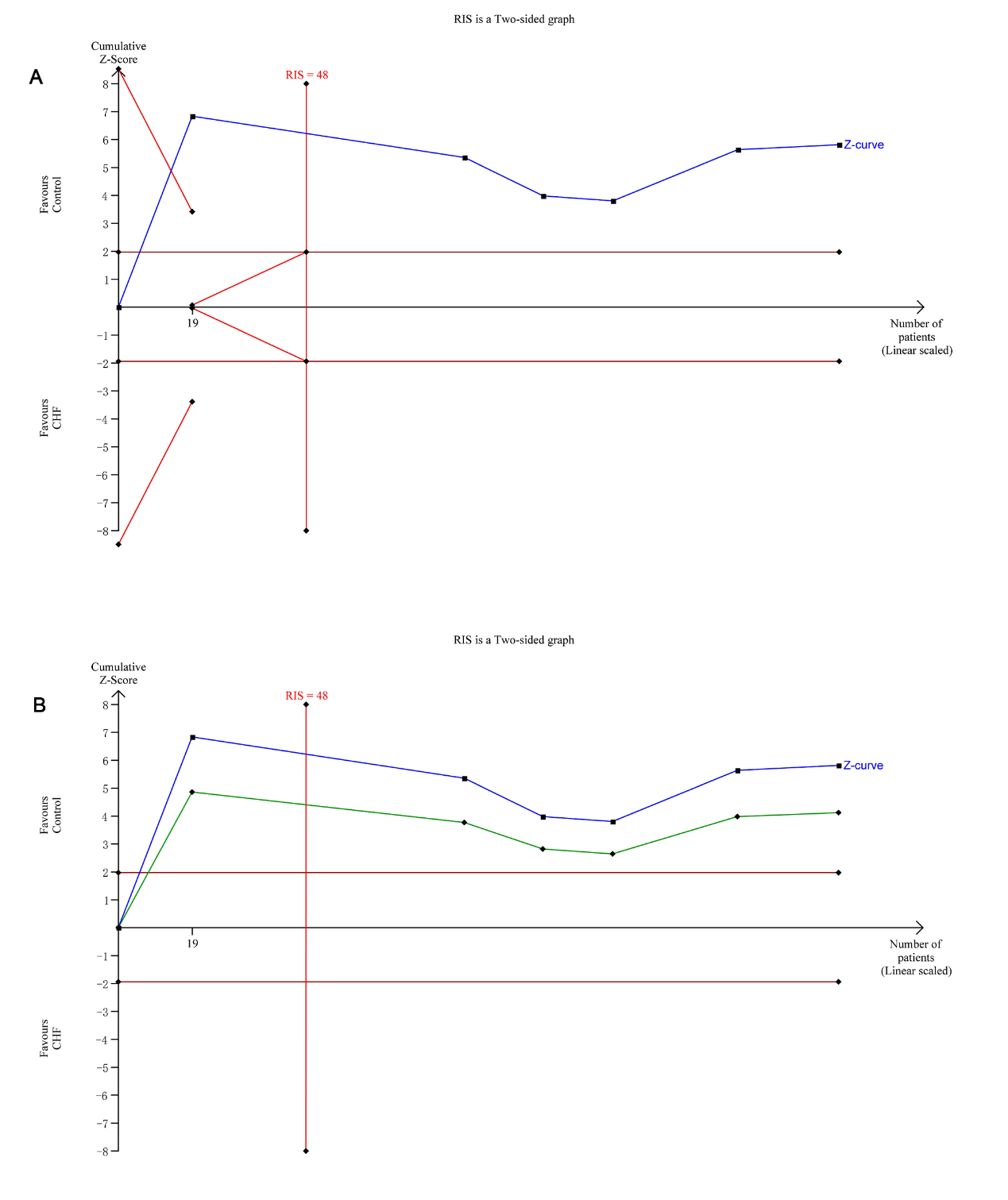


An estimated required information size (RIS) of 48 patients was calculated based on an anticipated relative risk reduction (RRR) of 13.7%, α=0.05 (two-sided), β=0.20 (power=80%). Based on a random-effect model, the blue z-curve crossed trial sequential monitoring boundary in the graph above (A) and the green z-curve after penalized tests crossed conventional boundary in the following graph (B).

**Fig. S4.** Trial sequential analysis for flow-mediated dilation between patients with heart failure with preserved ejection fraction and healthy participants.


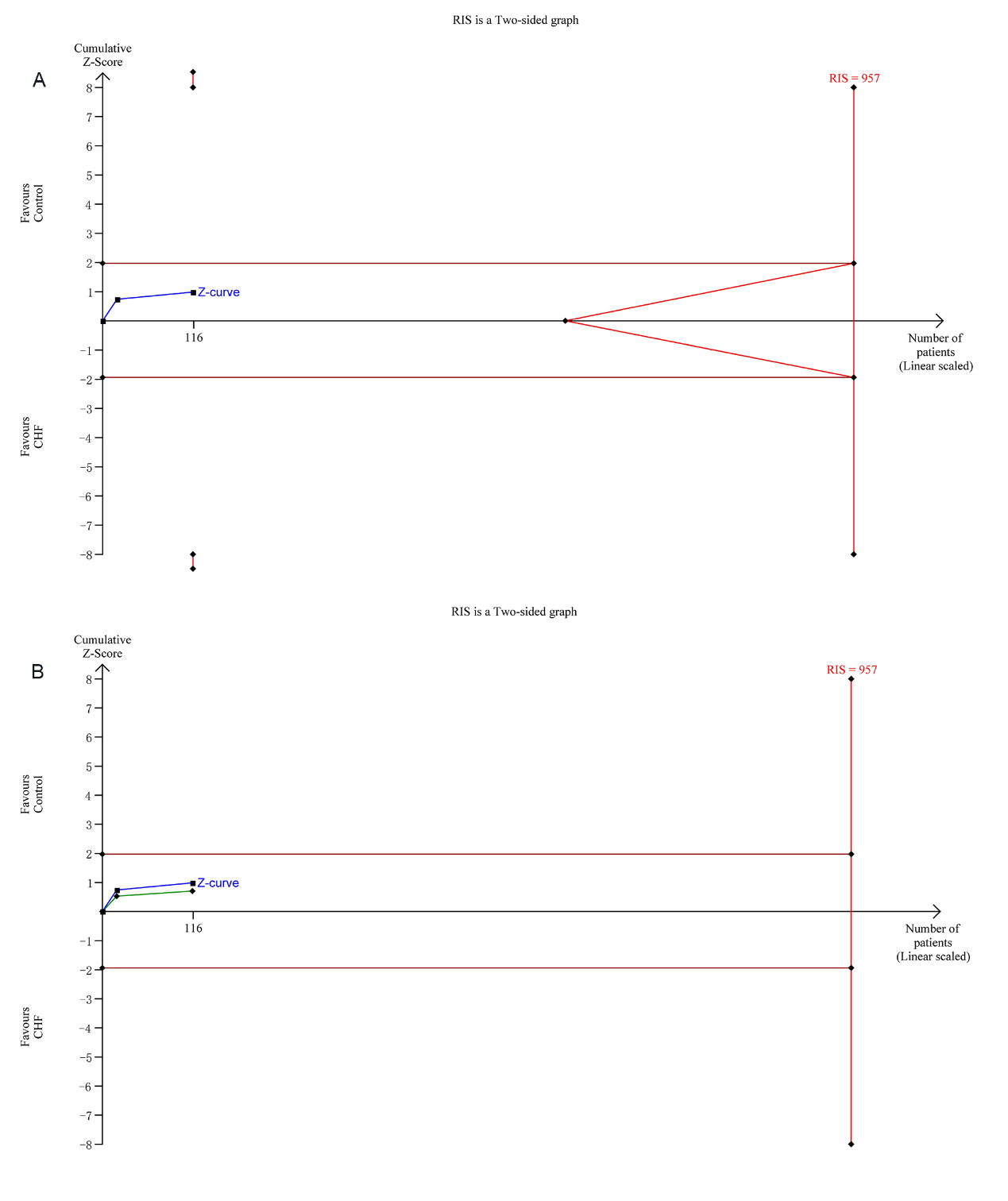


An estimated required information size (RIS) of 957 patients was calculated based on an anticipated relative risk reduction (RRR) of 13.7%, α=0.05 (two-sided), β=0.20 (power=80%). Based on a random-effect model, both the blue z-curve in the graph above (A) and the green z-curve after penalized tests in the following graph (B) did not cross conventional boundary.

**Fig. S5.** Trial sequential analysis for flow-mediated dilation in patients with chronic heart failure in the cases of exercise and non-exercise.


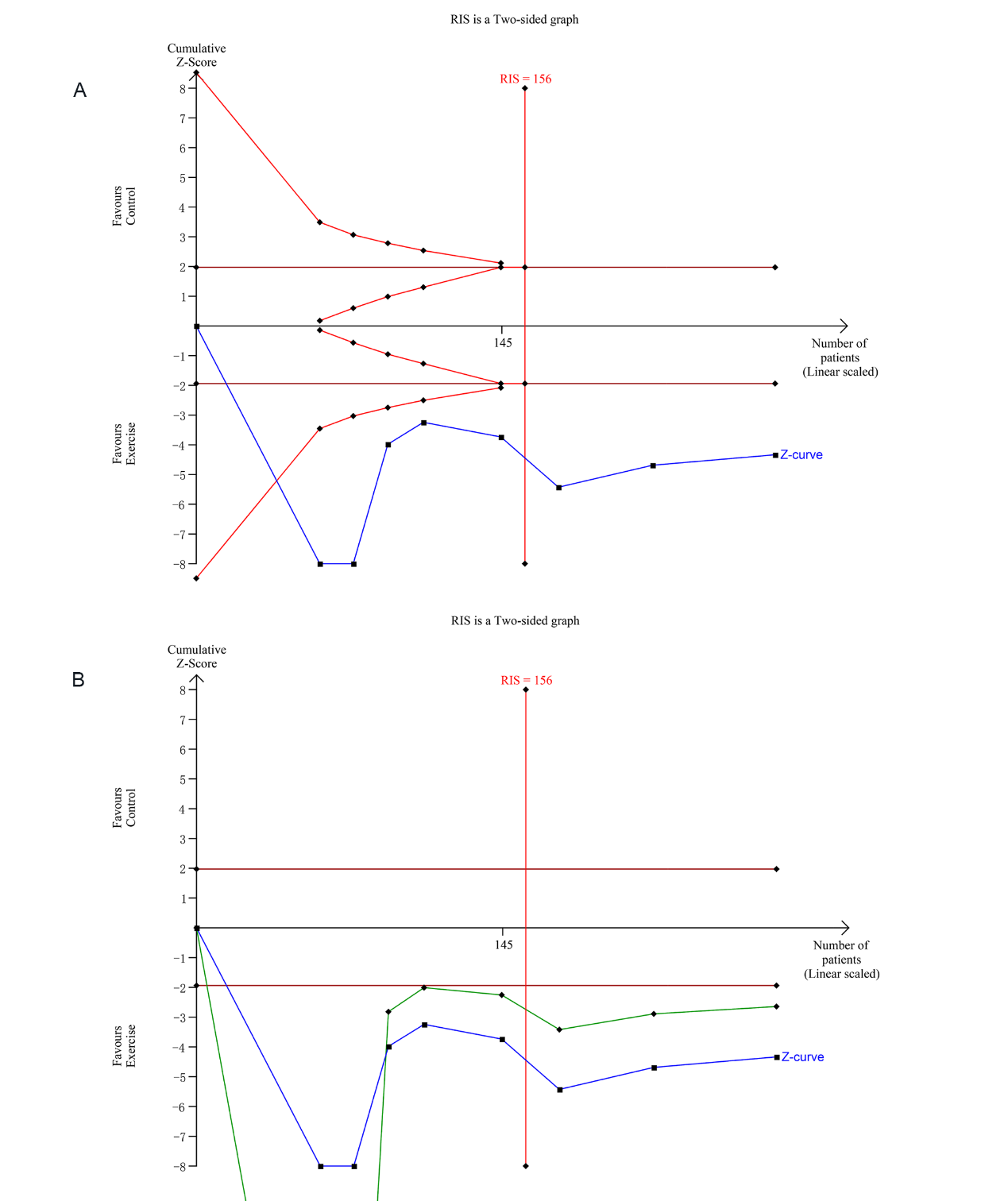


An estimated required information size (RIS) of 156 patients was calculated based on an anticipated relative risk reduction (RRR) of 13.7%, α=0.05 (two-sided), β=0.20 (power=80%). Based on a random-effect model, the blue z-curve crossed trial sequential monitoring boundary in the graph above (A) and the green z-curve after penalized tests crossed conventional boundary in the following graph (B).

**Fig. S6.** Trial sequential analysis for flow-mediated dilation in patients with heart failure with reduced ejection fraction in the cases of exercise and non-exercise.


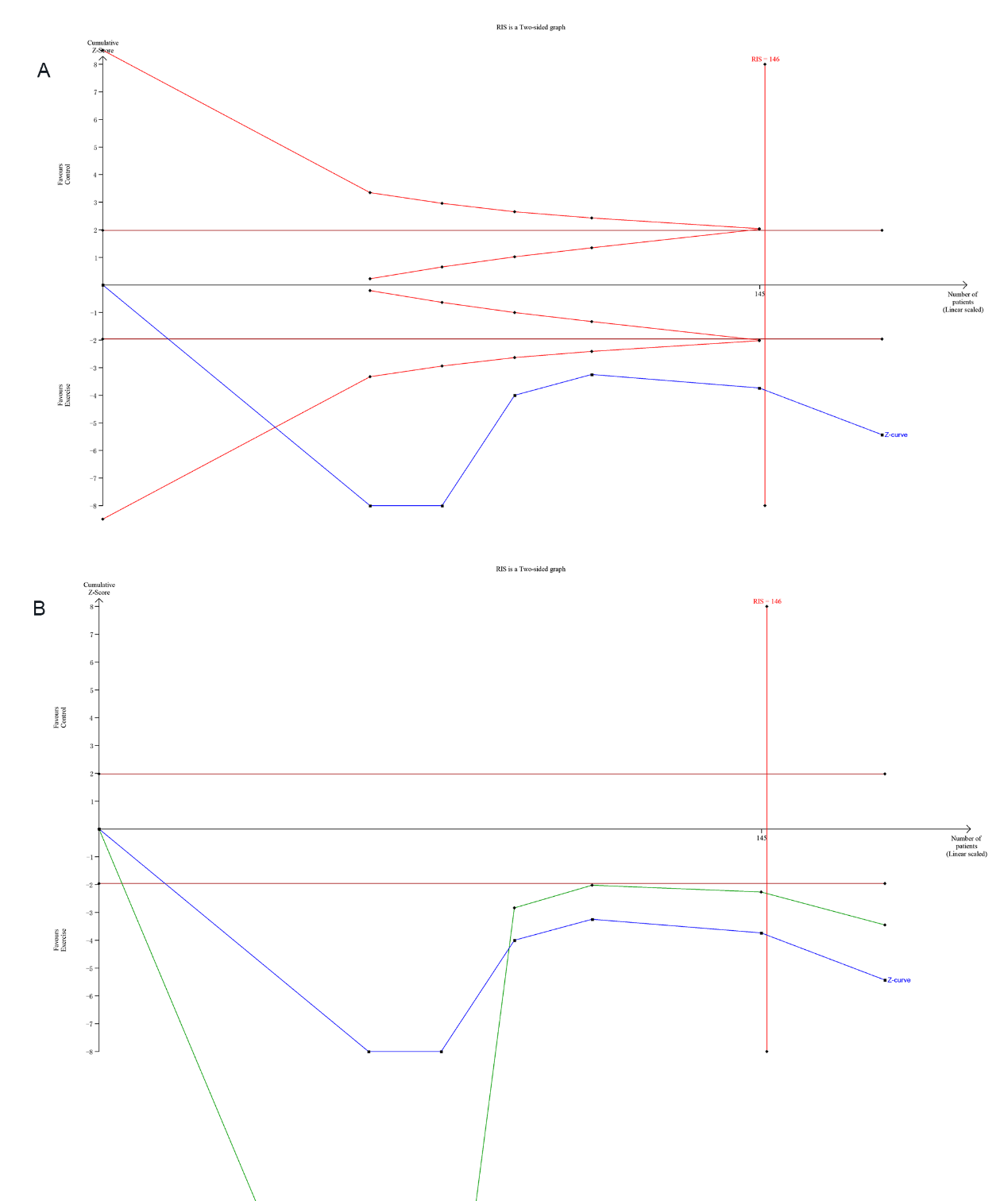


An estimated required information size (RIS) of 146 patients was calculated based on an anticipated relative risk reduction (RRR) of 13.7%, α=0.05 (two-sided), β=0.20 (power=80%). Based on a random-effect model, the blue z-curve crossed trial sequential monitoring boundary in the graph above (A) and the green z-curve after penalized tests crossed conventional boundary in the following graph (B).

**Fig. S7.** Trial sequential analysis for flow-mediated dilation in patients with heart failure with preserved ejection fraction in the cases of exercise and non-exercise.


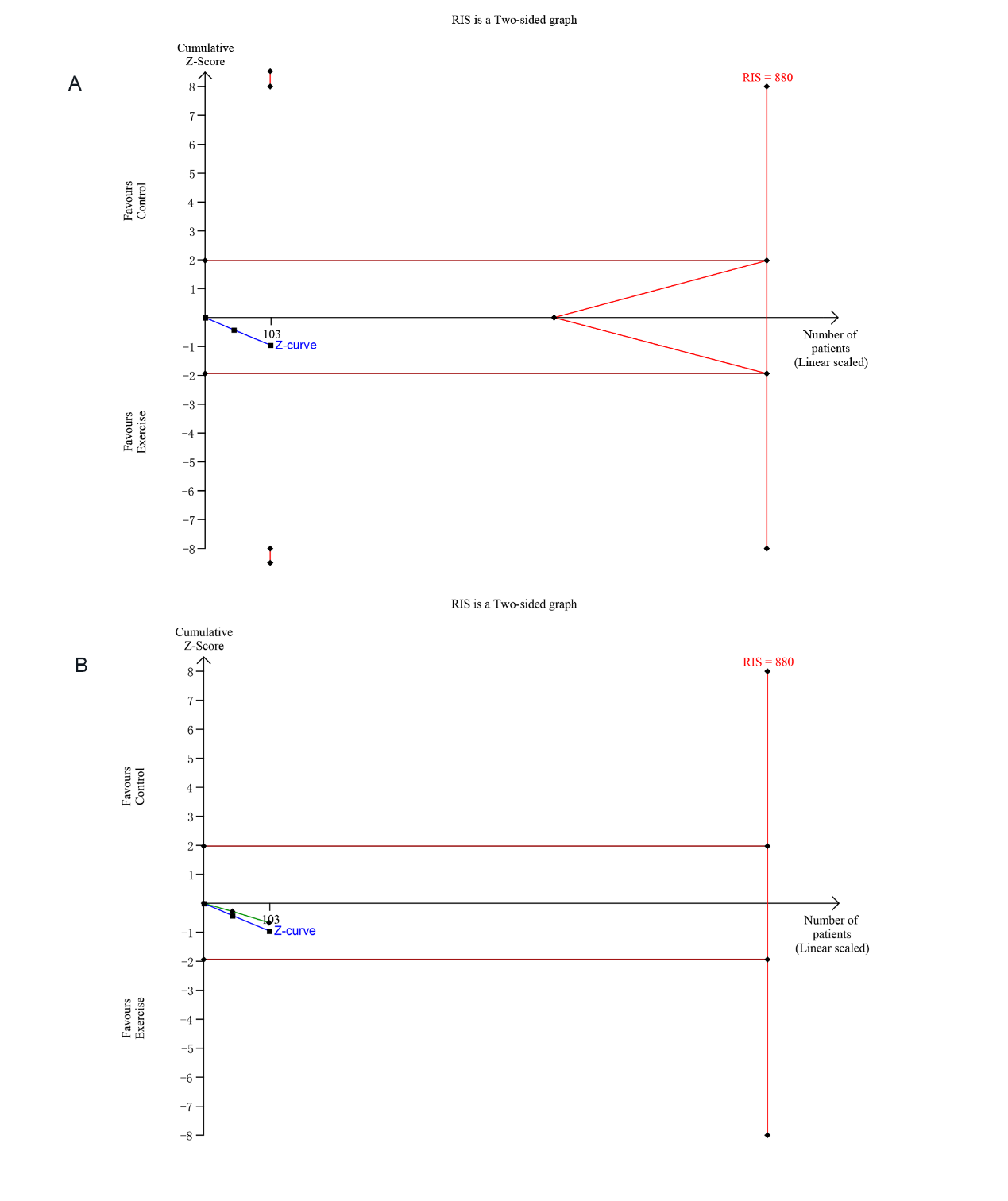


An estimated required information size (RIS) of 880 patients was calculated based on an anticipated relative risk reduction (RRR) of 13.7%, α=0.05 (two-sided), β=0.20 (power=80%). Based on a random-effect model, both the blue z-curve in the graph above (A) and the green z-curve after penalized tests in the following graph (B) did not cross conventional boundary.

**Fig. S8.** Trial sequential analysis for flow-mediated dilation in patients with chronic heart failure in the cases of moderate-intensity exercise training and non-exercise training.


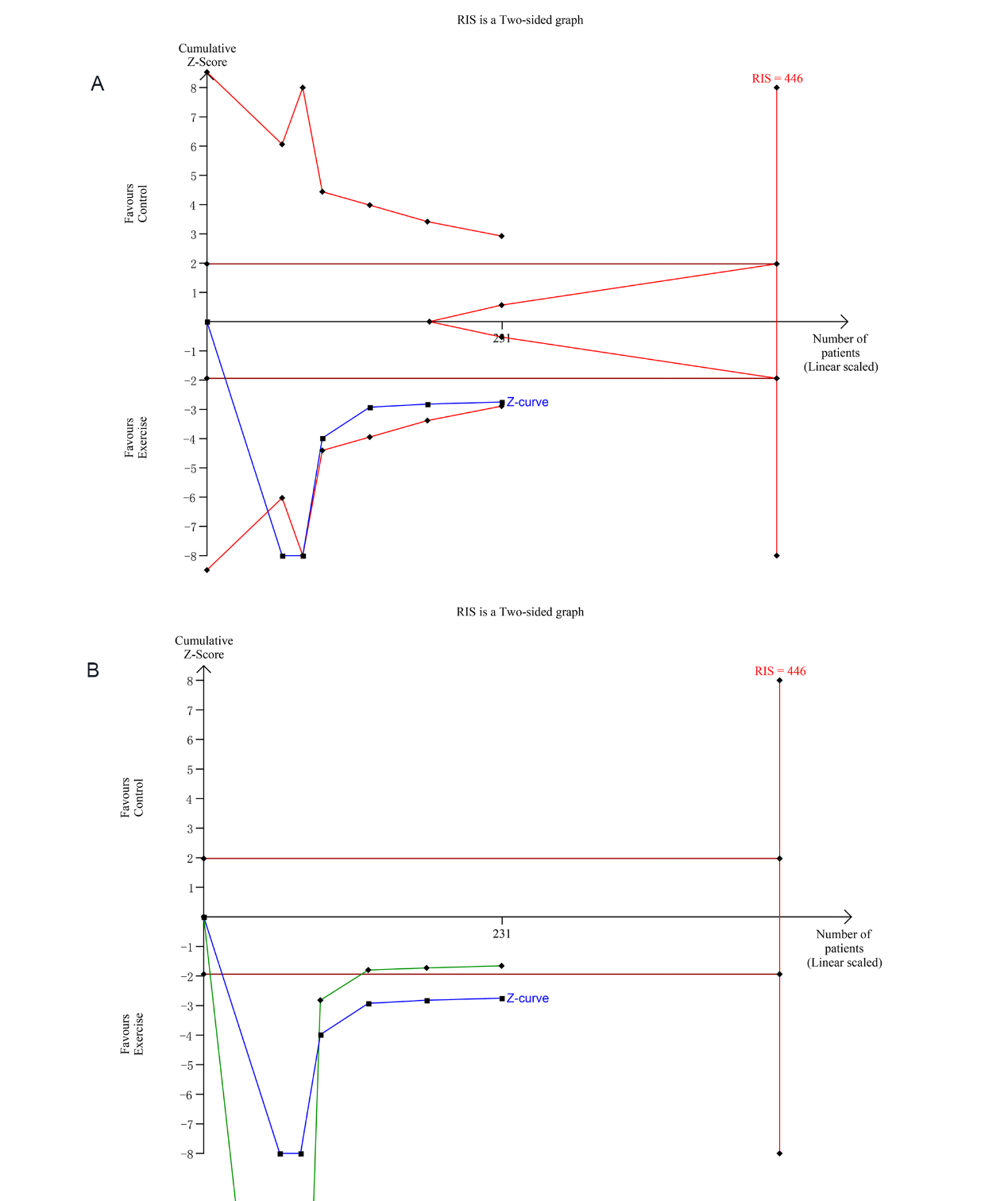


An estimated required information size (RIS) of 446 patients was calculated based on an anticipated relative risk reduction (RRR) of 13.7%, α=0.05 (two-sided), β=0.20 (power=80%). Based on a random-effect model, the blue z-curve crossed conventional boundary, however after two trials the z-curve went below trial sequential monitoring boundary again in the graph above (A) and the green z-curve after penalized tests went below conventional boundary again as well in the following graph (B). No firm conclusion can be drawn, and further high-quality studies are needed.

**Fig. S9.** Trial sequential analysis for flow-mediated dilation in patients with chronic heart failure in the cases of high-intensity exercise training and non-exercise training.
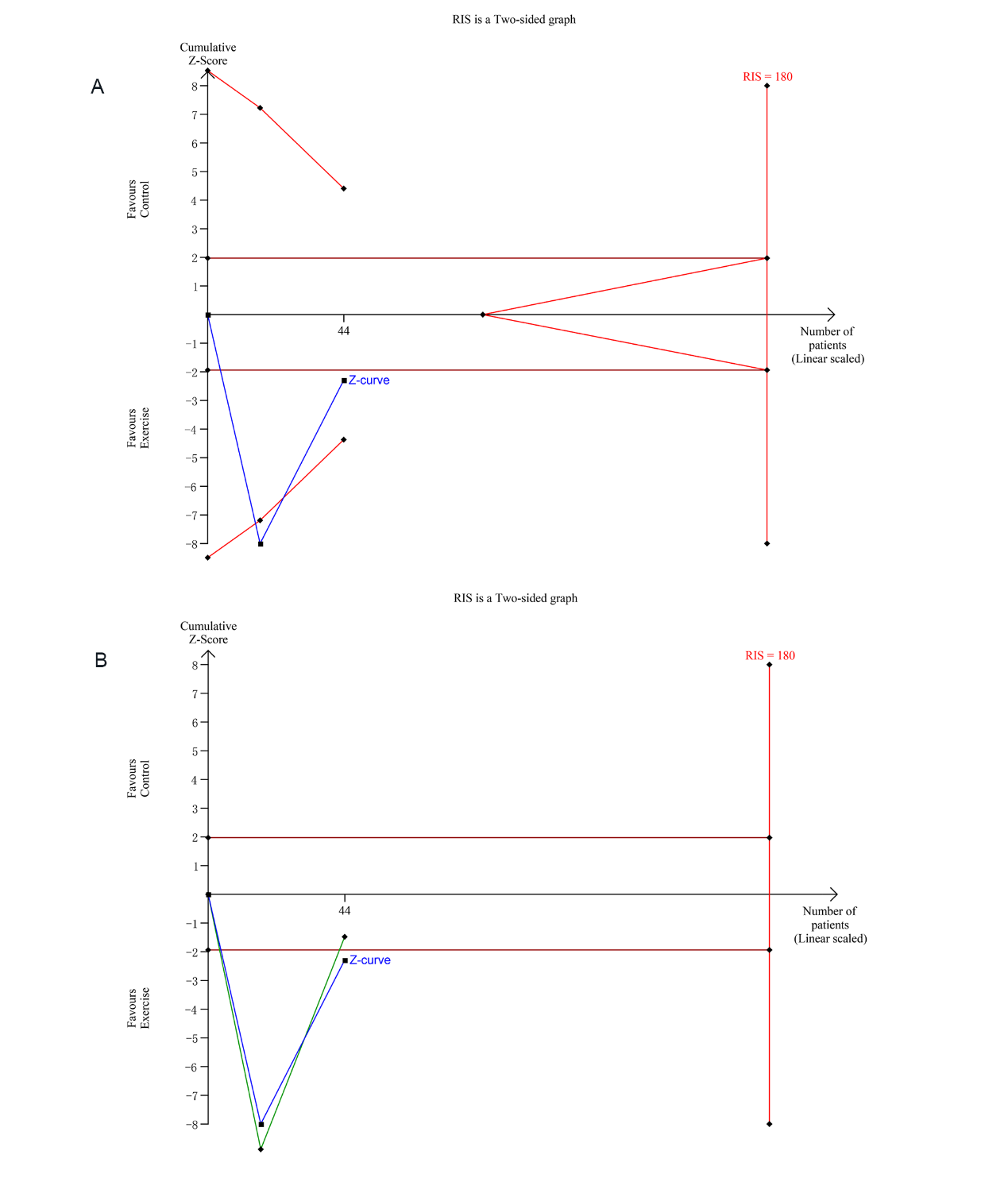


An estimated required information size (RIS) of 180 patients was calculated based on an anticipated relative risk reduction (RRR) of 13.7%, α=0.05 (two-sided), β=0.20 (power=80%). Based on a random-effect model, the blue z-curve crossed conventional boundary, however after one trial the z-curve went below trial sequential monitoring boundary again in the graph above (A) and the green z-curve after penalized tests went below conventional boundary again as well in the following graph (B). No firm conclusion can be drawn, and further high-quality studies are needed.

**Fig. S10.** Trial sequential analysis for flow-mediated dilation in patients with chronic heart failure in the cases of high-intensity exercise training and moderate-intensity exercise training.


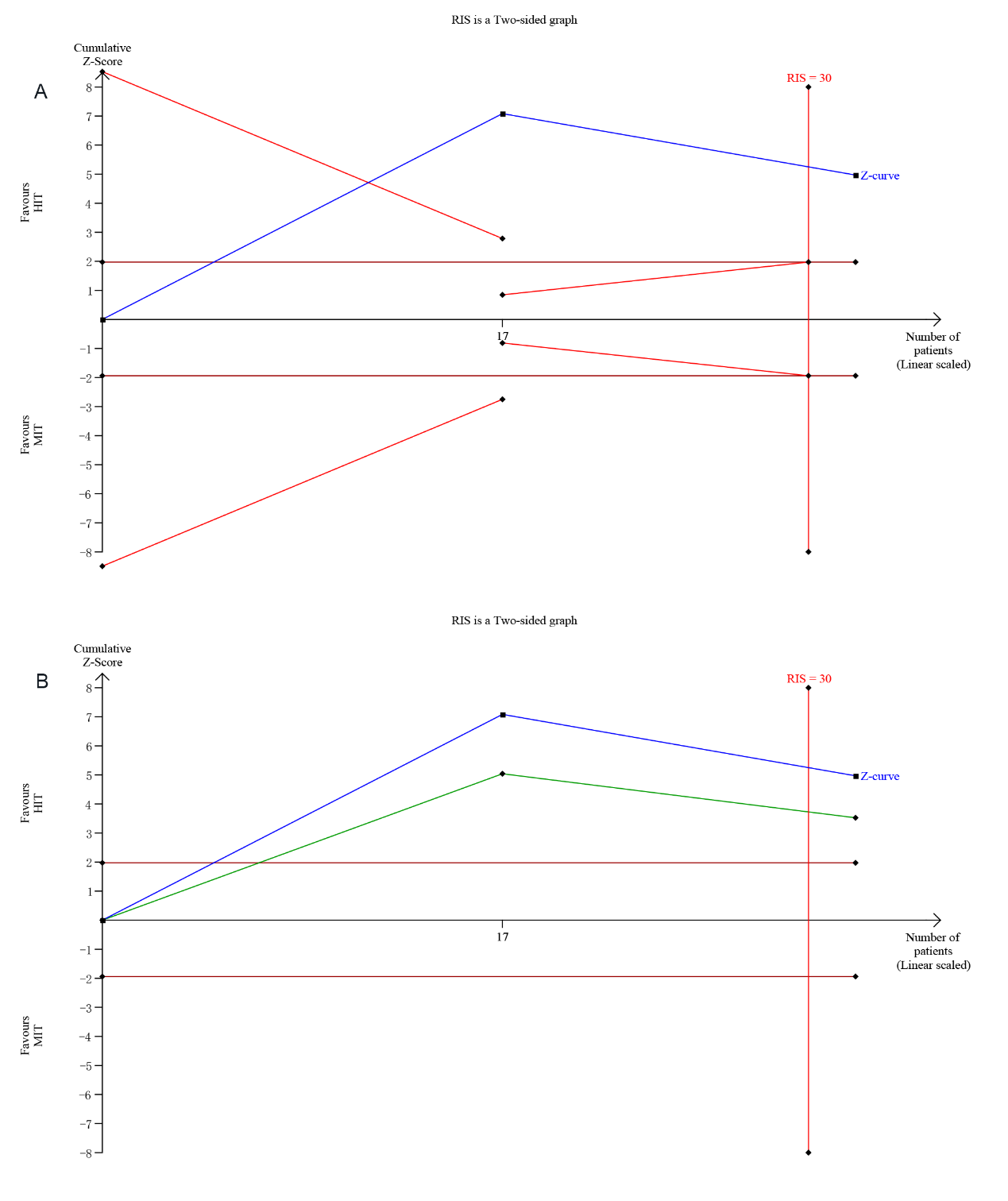


An estimated required information size (RIS) of 30 patients was calculated based on an anticipated relative risk reduction (RRR) of 13.7%, α=0.05 (two-sided), β=0.20 (power=80%). Based on a random-effect model, the blue z-curve crossed trial sequential monitoring boundary in the graph above (A) and the green z-curve after penalized tests crossed conventional boundary in the following graph (B).

**Fig. S11.** Trial sequential analysis for augmentation Index between patients with chronic heart failure and healthy participants.


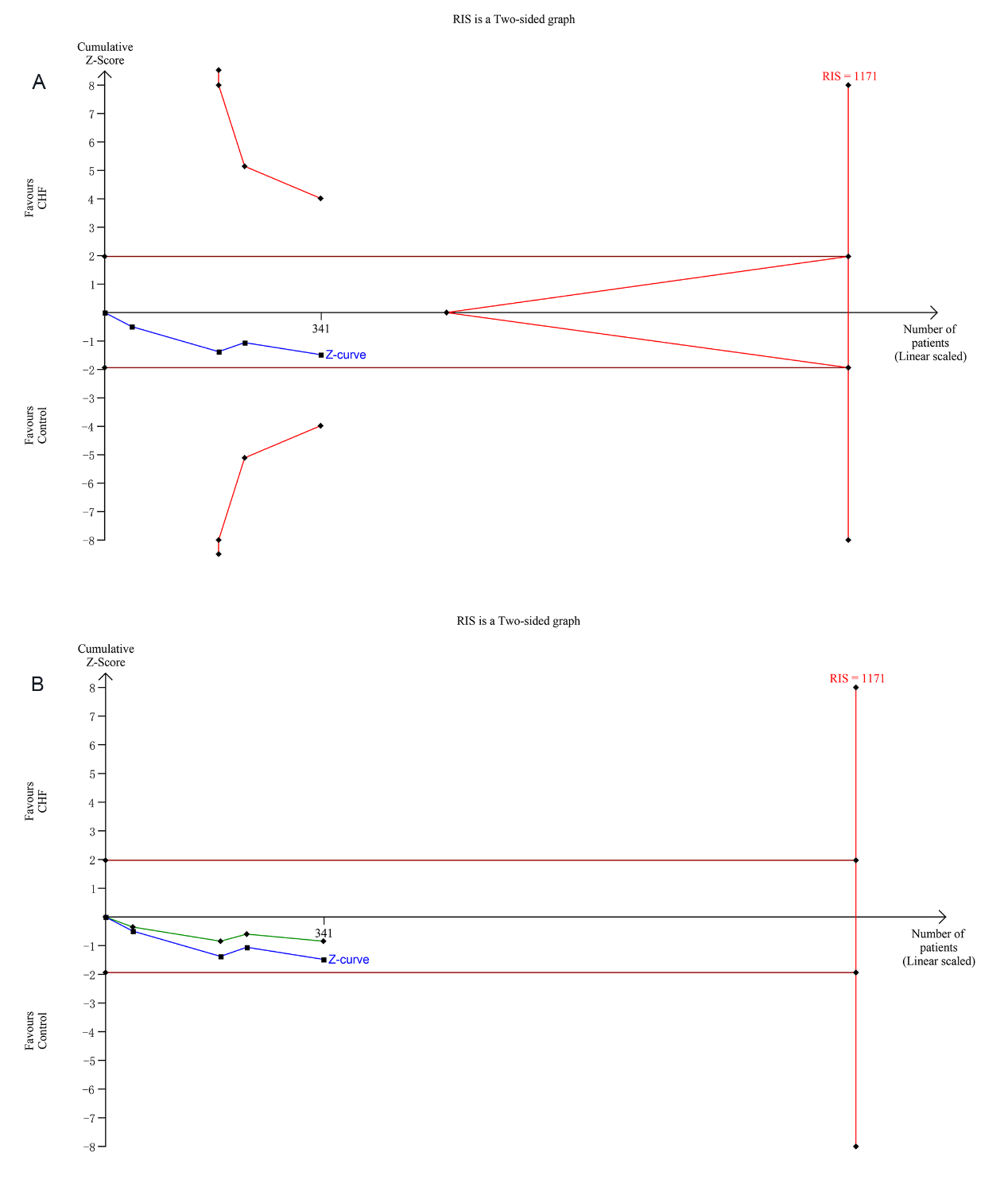


An estimated required information size (RIS) of 1171 patients was calculated based on an anticipated relative risk reduction (RRR) of 13.7%, α=0.05 (two-sided), β=0.20 (power=80%). Based on a random-effect model, both the blue z-curve in the graph above (A) and the green z-curve after penalized tests in the following graph (B) did not cross conventional boundary.

**Fig. S12.** Trial sequential analysis for augmentation Index between patients with heart failure with preserved ejection fraction and healthy participants.


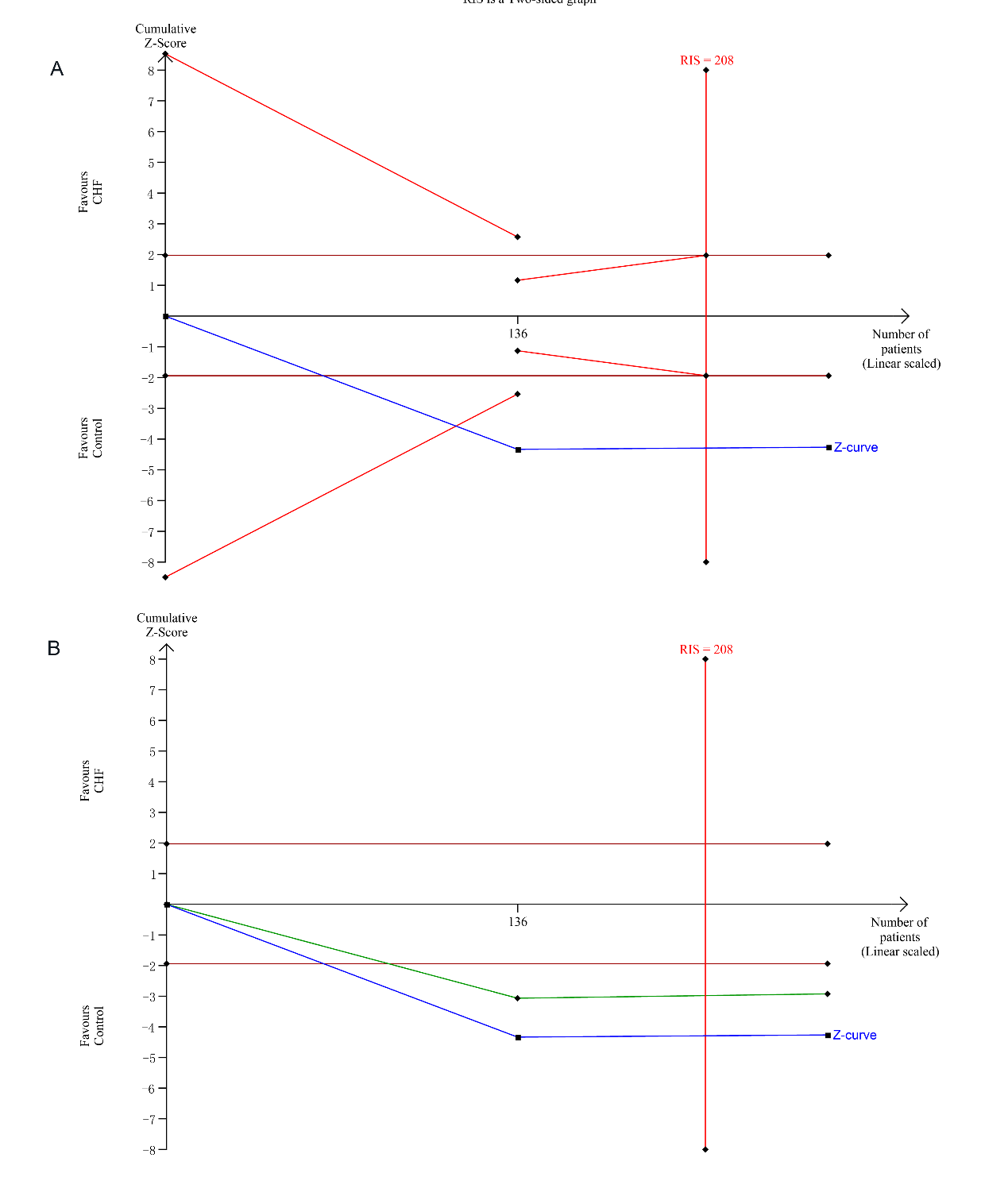


An estimated required information size (RIS) of 208 patients was calculated based on an anticipated relative risk reduction (RRR) of 13.7%, α=0.05 (two-sided), β=0.20 (power=80%). Based on a random-effect model, the blue z-curve crossed trial sequential monitoring boundary in the graph above (A) and the green z-curve after penalized tests crossed conventional boundary in the following graph (B).

**Fig. S13.** Trial sequential analysis for augmentation Index between patients with heart failure with reduced ejection fraction and healthy participants.


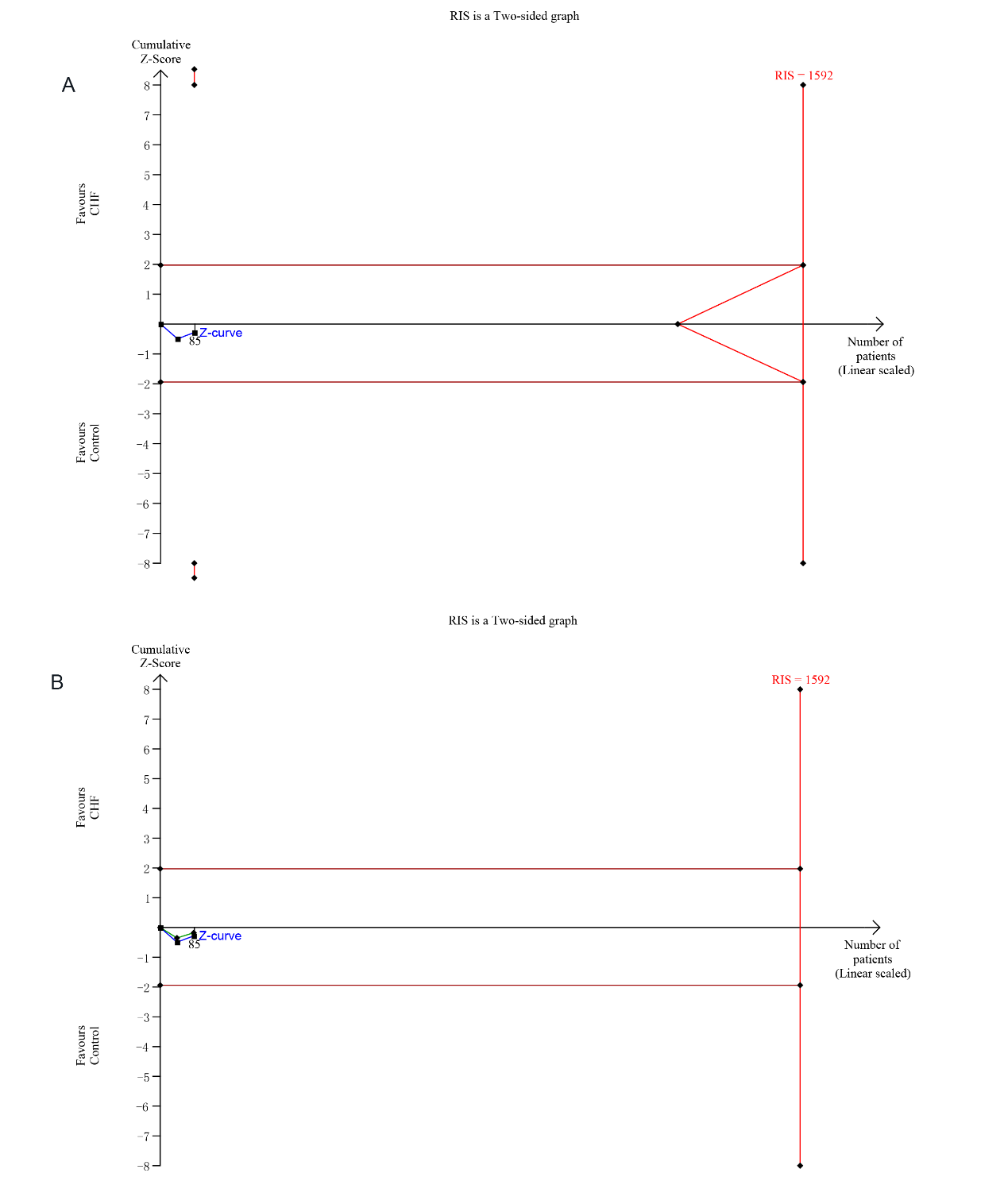


An estimated required information size (RIS) of 1592 patients was calculated based on an anticipated relative risk reduction (RRR) of 13.7%, α=0.05 (two-sided), β=0.20 (power=80%). Based on a random-effect model, both the blue z-curve in the graph above (A) and the green z-curve after penalized tests in the following graph (B) did not cross conventional boundary.

**Fig. S14 .** Sensitivity analysis of flow-mediated dilation in patients with chronic heart failure and healthy participants.


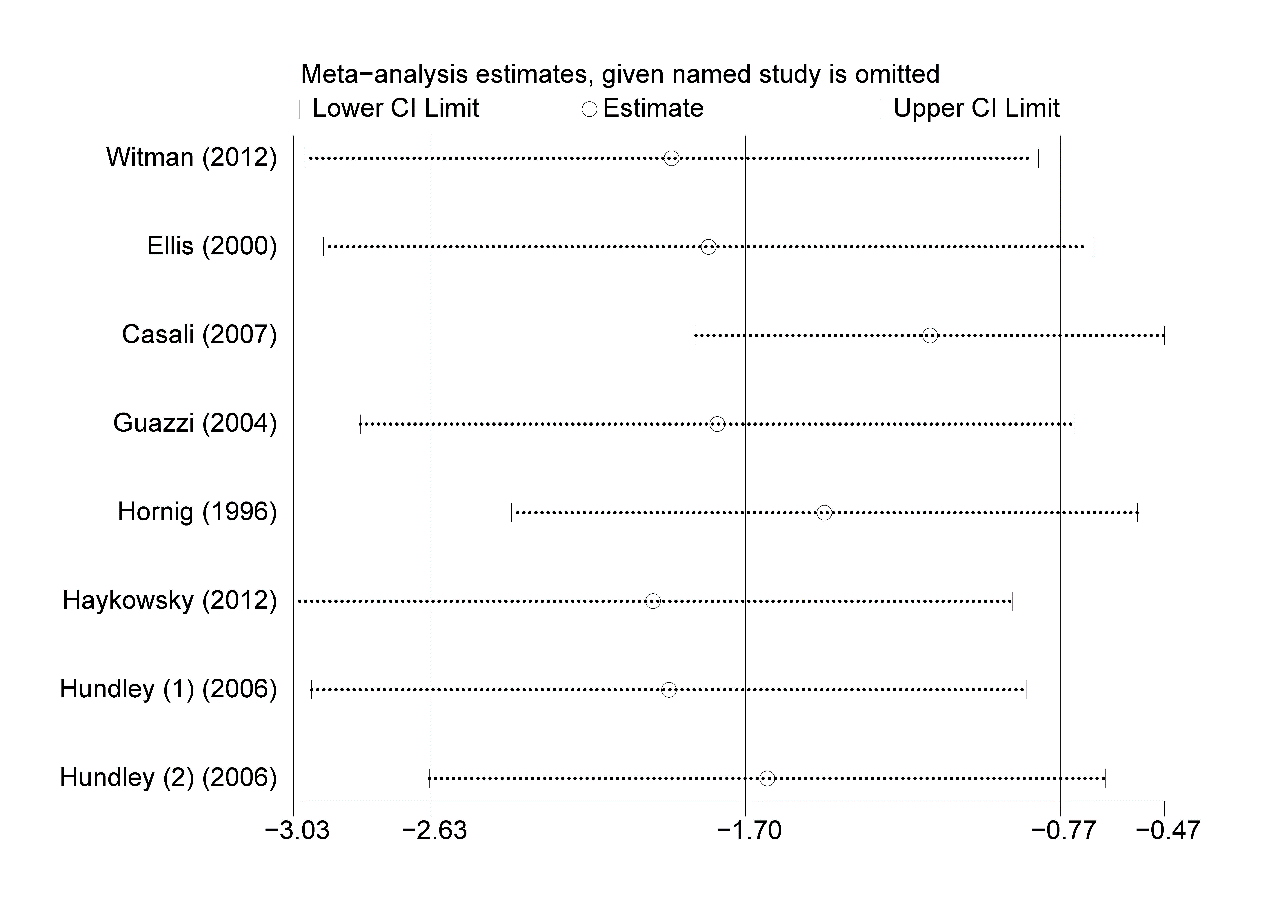


**Fig. S15.** Sensitivity analysis of augmentation index in patients with chronic heart failure and healthy participants.


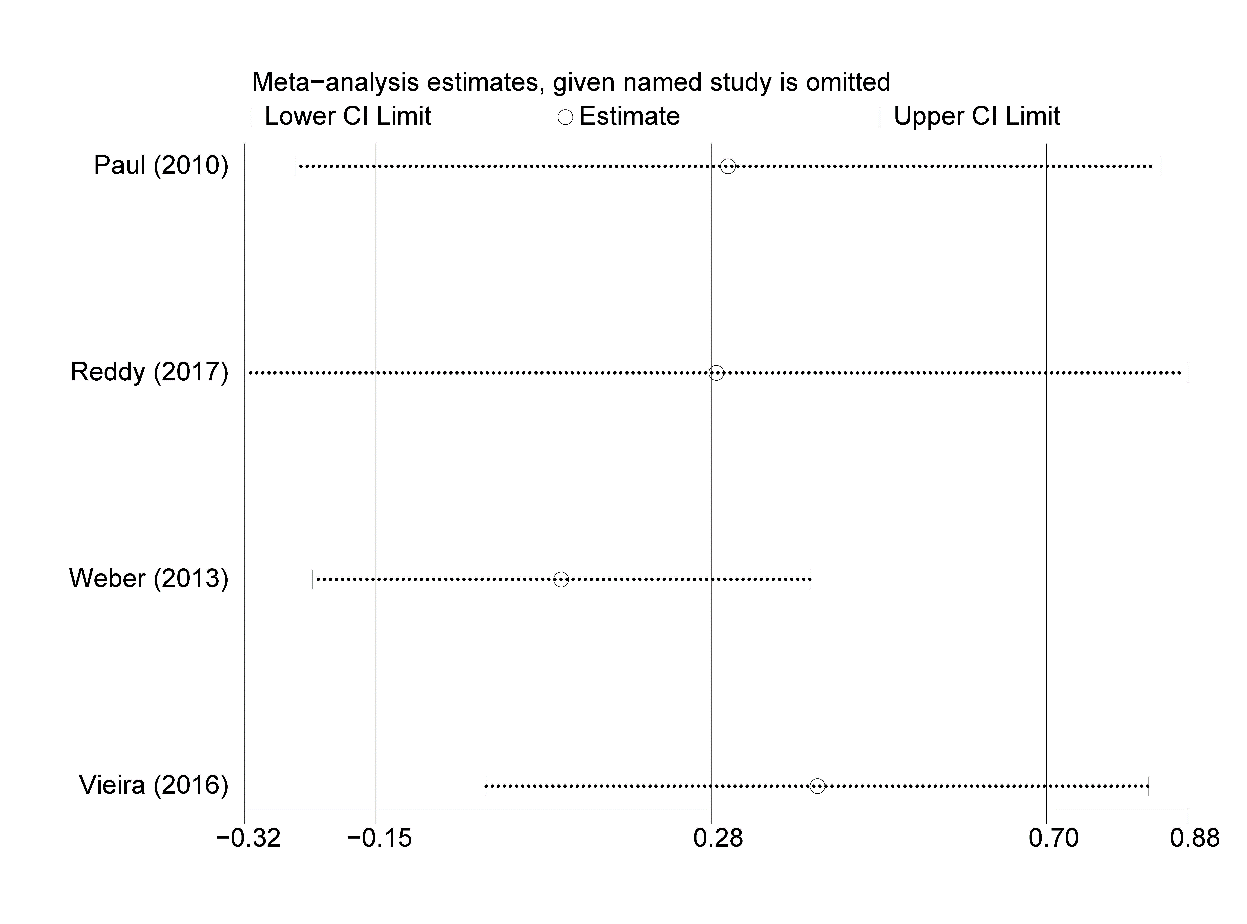


**Fig. S16.** Sensitivity analysis of flow-mediated dilation in patients with chronic heart failure in the cases of exercise and non-exercise.


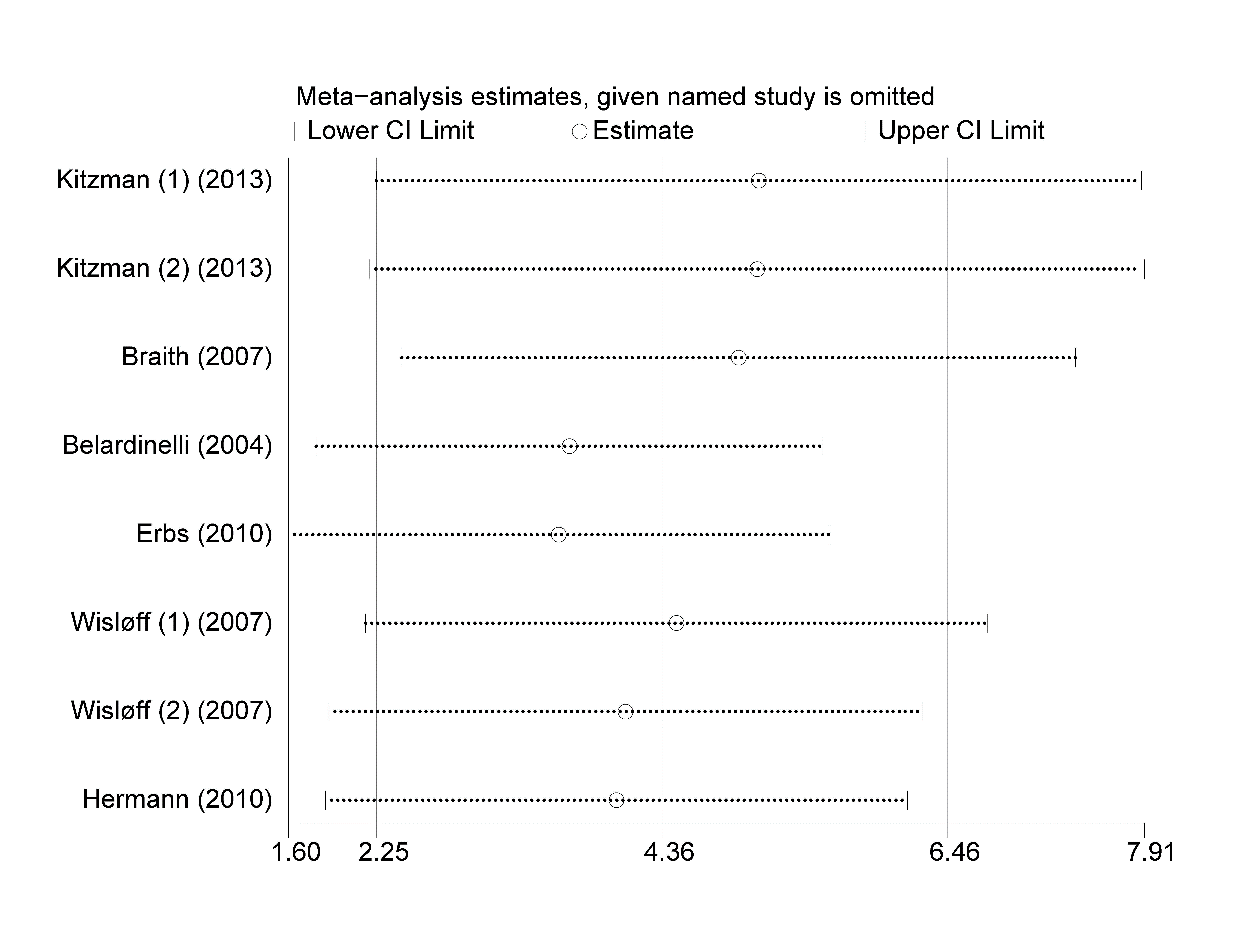

Supplement: Supplementary file 1 — Additional file 1: Fig. S1. Risk of bias of all included articles. Fig. S2. Trial sequential analysis for flow-mediated dilation between patients with chronic heart failure and healthy participants. Fig. S3. Trial sequential analysis for flow-mediated dilation between patients with heart failure with reduced ejection fraction and healthy participants. Fig. S4. Trial sequential analysis for flow-mediated dilation between patients with heart failure with preserved ejection fraction and healthy participants. Fig. S5. Trial sequential analysis for flow-mediated dilation in patients with chronic heart failure in the cases of exercise and non-exercise. Fig. S6. Trial sequential analysis for flow-mediated dilation in patients with heart failure with reduced ejection fraction in the cases of exercise and non-exercise. Fig. S7. Trial sequential analysis for flow-mediated dilation in patients with heart failure with preserved ejection fraction in the cases of exercise and non-exercise. Fig. S8. Trial sequential analysis for flow-mediated dilation in patients with chronic heart failure in the cases of moderate-intensity exercise training and non-exercise training. Fig. S9. Trial sequential analysis for flow-mediated dilation in patients with chronic heart failure in the cases of high-intensity exercise training and non-exercise training. Fig. S10. Trial sequential analysis for flow-mediated dilation in patients with chronic heart failure in the cases of high-intensity exercise training and moderate-intensity exercise training. Fig. S11. Trial sequential analysis for augmentation Index between patients with chronic heart failure and healthy participants. Fig. S12. Trial sequential analysis for augmentation Index between patients with heart failure with preserved ejection fraction and healthy participants. Fig. S13. Trial sequential analysis for augmentation Index between patients with heart failure with reduced ejection fraction and healthy participants. Fig. S14. Sensitivity a [file 12872_2022_2792_MOESM1_ESM.docx]
